# Geometric morphometrics and paleoproteomics enlighten the paleodiversity of *Pongo*

**DOI:** 10.1371/journal.pone.0291308

**Published:** 2023-12-15

**Authors:** Jülide Kubat, Ryan Paterson, Ioannis Patramanis, Graeme Barker, Fabrice Demeter, Arnaud Filoux, Ottmar Kullmer, Meaghan Mackie, Tomas Marques-Bonet, Nguyen Thi Mai Huong, Nguyen Anh Tuan, Sytha Pheng, Jessica Rippengal, Friedemann Schrenk, Viengkeo Souksavatdy, Lim Tze Tshen, Athiwat Wattanapituksakul, Wei Wang, Clément Zanolli, Enrico Cappellini, Anne-Marie Bacon

**Affiliations:** 1 CNRS, BABEL, Université Paris Cité, Paris, France; 2 Department of Palaeoanthropology, Senckenberg Research Institute and Natural History Museum Frankfurt, Frankfurt am Main, Germany; 3 Globe Institute, University of Copenhagen, Copenhagen, Denmark; 4 McDonald Institute for Archaeological Research, University of Cambridge, Cambridge, United Kingdom; 5 Lundbeck Foundation GeoGenetics Centre, Globe Institute, University of Copenhagen, Copenhagen, Denmark; 6 UMR 7206 Eco Anthropologie, Muséum National d’Histoire Naturelle, CNRS, Paris, France; 7 Palaeontological Research and Education Centre, Mahasarakham University, Mahasarakham, Thailand; 8 The Novo Nordisk Foundation Center for Protein Research, University of Copenhagen, Copenhagen, Denmark; 9 Institute of Evolutionary Biology (UPF-CSIC), PRBB, Barcelona, Spain; 10 Catalan Institution of Research and Advanced Studies (ICREA), Passeig de Lluís Companys, Barcelona, Spain; 11 CNAG-CRG, Centre for Genomic Regulation (CRG), Barcelona Institute of Science and Technology (BIST), Barcelona, Spain; 12 Institut Català de Paleontologia Miquel Crusafont, Universitat Autònoma de Barcelona, Barcelona, Spain; 13 Anthropological and Palaeoenvironmental Department, Institute of Archaeology, Ha Noi, Vietnam; 14 Royal University of Fine Arts, Phnom Penh, Cambodia; 15 Department of Heritage, Ministry of Information, Culture and Tourism, Vientiane, Lao People’s Democratic Republic; 16 Department of Geology, Faculty of Science, Universiti Malaya, Kuala Lumpur, Malaysia; 17 Institute of Cultural Heritage, Shandong University, Qingdao, China; 18 CNRS, MCC, PACEA, UMR 5199, Univ. Bordeaux, Pessac, France; Griffith University, AUSTRALIA

## Abstract

Pleistocene *Pongo* teeth show substantial variation in size and morphology, fueling taxonomic debates about the paleodiversity of the genus. We investigated prominent features of the enamel-dentine-junction junction (EDJ)–phylogenetically informative internal structures–of 71 fossil *Pongo* lower molars from various sites by applying geometric morphometrics and conducted paleoproteomic analyses from enamel proteins to attempt to identify extinct orangutan species. Forty-three orangutan lower molars representing *Pongo pygmaeus* and *Pongo abelii* were included for comparison. The shape of the EDJ was analyzed by placing five landmarks on the tip of the main dentine horns, and 142 semilandmarks along the marginal ridges connecting the dentine horns. Paleoproteomic analyses were conducted on 15 teeth of Late Pleistocene *Pongo* using high-resolution tandem mass spectrometry. The geometric morphometric results show variations in EDJ shape regarding aspects of the height and position of the dentine horns and connecting ridges. Despite the issue of molar position and sample size, modern molars are distinguished from fossil counterparts by their elongated tooth outline and narrowly positioned dentine horns. Proteomic results show that neither a distinction of *P*. *pygmaeus* and *P*. *abelii*, nor a consistent allocation of fossil specimens to extant species is feasible. Based on the EDJ shape, the (late) Middle to Late Pleistocene *Pongo* samples from Vietnam share the same morphospace, supporting the previous allocation to *P*. *devosi*, although substantial overlap with Chinese fossils could also indicate close affinities with *P*. *weidenreichi*. The hypothesis that both species represent one chronospecies cannot be ruled out. Two fossil specimens, one from Tam Hay Marklot (Laos, Late Pleistocene), and another from Sangiran (Java, Early to Middle Pleistocene), along with some specimens within the Punung sample (Java), exhibit affinities with *Pongo abelii*. The Punung fossils might represent a mix of early Late Pleistocene and later specimens (terminal Pleistocene to Holocene) related to modern *Pongo*. The taxonomy and phylogeny of the complete Punung sample needs to be further investigated.

## 1. Introduction

A large geographical and temporal distribution of extinct orangutans (*Pongo*) is evidenced by the rich Early to Late Pleistocene fossil record ranging from southern China and continental Southeast Asia (Vietnam, Laos, Cambodia, Thailand, Peninsular Malaysia) to insular Southeast Asia–Sumatra, Java, and Borneo [1–5]. In a recent revision of the Pleistocene *Pongo* species diversity, Harrison et al. [6, 7] proposed to assign all Early to Late Pleistocene specimens from southern China and Vietnam to *Pongo weidenreichi*, encompassing in one single species all taxa defined previously [3]. Late Pleistocene specimens from the Sibrambang in the Padang highlands (Central Sumatra) were recognized as *Pongo palaeosumatrensis* [1], whereas the Late Pleistocene orangutans from Niah Cave (Sarawak, Malaysian Borneo) were assigned to the modern species *Pongo pygmaeus* [8–10]. Other extinct samples from Indonesia were tentatively attributed to *Pongo javensis* from the Late Pleistocene site of Punung (Central Java) and *Pongo duboisi* from the Late Pleistocene Lida Ayer and Djambu sites in the Padang highlands (Central Sumatra) byHarrison [6] based on Drawhorn’s work [11].

Extant species can be largely distinguished based on several features related to hair color, cheek flanges, various cranial features, DNA, and geographical distribution [6, 12–17]. To date, only three *Pongo* species remain in the Ponginae subfamily, namely *P*. *abelii* and *P*. *tapanuliensis* in Sumatra, and *P*. *pygmaeus* in Borneo [5, 17–20]. The reasons for this impoverishment of diversity and decline in geographical distribution of *Pongo* are linked to the loss of suitable habitats caused by multiple factors such as climatic change during the Pleistocene and anthropic impacts such as hunting and deforestation [21]. The slow reproduction rates and prolonged dependence of infants on their mothers makes them additionally vulnerable when facing these impacts [22]. This might also have caused the decrease in *Pongo* populations in the Holocene.

Soft tissue, hair, and/or DNA have not been recovered so far in fossil orangutans and cranial remains are rare. In the absence of these features, the most diagnostic elements in the fossil records are teeth. However, it was proposed that mixed samples of isolated teeth of extant species such as *P*. *abelii* and *P*. *pygmaeus* would be impossible to separate from each other solely based on their external morphology [6]. Therefore, if even well-identified extant species are difficult to separate based on dental traits, the validity of fossil *Pongo* species identification raises questions, given that most fossilized remains are isolated teeth. Furthermore, occlusal wear during the lifetime of an individual and post-mortem taphonomy can alter the shape of the crown, which makes it especially difficult to identify species based on the macromorphology of molars [23, 24].

Extinct *Pongo* exhibits a high degree of variation in tooth size and morphology [5, 6]. Several attempts to distinguish taxa at the (sub)species level have been proposed based on odontometric measurements and non-metric features recorded on teeth [3, 6, 7]. Criteria such as relative tooth size, degree of crenulation on the occlusal surfaces of molars and premolars, size of dental crown areas, and variability of buccolingual and mesiodistal tooth dimensions, have been applied to identify morphotypes relating to paleospecies [1, 3, 6, 7, 11, 25]. However, any of these criteria cannot be used as secure diagnostic features due to the continuous gradient of these traits among fossil specimens and/or to the large morphodimensional overlap between *Pongo* samples [5].

Hence, the characterization of these species may significantly benefit from geometric morphometrics of internal structures like the enamel-dentine-junction (EDJ). The EDJ is protected by the enamel and, therefore, generally less affected by taphonomic damage and occlusal wear than the surface of the crown [23, 24, 26, 27]. This structure has also been recognized as a reliable taxonomic proxy, even at the subspecies level [23, 26, 27].

In addition, paleoproteomic analyses of dental enamel can be performed as a complementary approach. Ancient proteins from fossil tooth enamel are proven to clarify species identifications and phylogenetic relationships, notably when ancient DNA is not preserved or accessible [28–35]. Proteins are more resistant to diagenesis than DNA and recent analyses prove that they can be successfully retrieved from dental enamel of Early Pleistocene fossils [36, 37].

Here, we applied geometric morphometrics on 71 mandibular molars of *Pongo* (Table 1 and S1 Table) from various Pleistocene sites and museum collections (Punung and Sangiran from Indonesia; Lang Trang, Tham Om, Hang Hum, Mai Da Dieu, Duoi U’Oi from Vietnam; Tam Hay Marklot and Tam Hang South from Laos; Ganxian from China; Chinese Apothecary collection housed at the Senckenberg Research Institute and Museum, Frankfurt). In addition, 43 modern *Pongo* mandibular molars were included in the dataset with both species *P*. *pygmaeus* and *P*. *abelii* represented. Paleoproteomics of dental enamel was performed on 15 Late Pleistocene teeth from Vietnam, Laos, Malaysia, and Thailand. Four of these specimens are mandibular molars and thus included in the geometric morphometric analyses.

**Table 1:** Fossil specimens included in the geometric morphometric analyses.

Region	Fossil site	Age	n	Species
China	Chinese Apothecary Collection	uncertain	4	*Sinanthropus officinalis* (von Koenigswald 1952) [40] *Pongo* sp. (Smith et al. 2018) [26]
	Ganxian Cave	362 ± 78 ka to 168.9 ± 2.4 ka	5	*P*. *weidenreichi* (Liang et al. 2022) [38]
	Tham Om	300–200 ka	8	*P*. *pygmaeus* spp. (Kha 1977) [60] *P*. *pygmaeus fromageti* (Schwartz et al. 1995) [3] *P*. *weidenreichi* (Harrison et al. 2014) [6]
	Lang Trang	80 ka or 480–146 ka	10	*P*. *pygmaeus* ssp. (Ciochon and Olsen 1991) [42] *Pongo pygmaeus ciochoni* (Schwartz et al. 1995) [3] *P*. *weidenreichi* (Harrison et al. 2014) [6]
Vietnam	Duoi U’Oi	70–60 ka	4	*P*. *pygmaeus* (Bacon et al. 2008, 2015) [45, 50]
	Hang Hum	140–80 ka	2	*P*. *pygmaeus pygmaeus* (Cuong 1992) [44] *P*. *pygmaeus devosi* (Schwartz et al. 1995) [3] *P*. *devosi* (Harrison et al. 2014) [6]
	Thung Lang	uncertain	10	*P*. *pygmaeus (Symia satyrus)* (Fromaget 1941) [47]
	Mai Da dieu	24–30 ka	1	*Pongo* sp. (Nguyen 2005) [46]
Indonesia	Sangiran Dome	1.5 Ma to 500 ka	3	*Pongo* sp. (Von Koenigswald 1940) [61]
	Punung	128–117 ka	22	*Pongo pygmaeus* (Badoux 1959, de Vos 1983) [56, 62] *P*. *pygmaeus javensis* (Drawhorn 1995) [11] *P*. *javensis* (Harrison et al. 2014) [6]
Laos	Tam Hay Marklot	38.4–13.5 ka	1	*Pongo* sp. (Bourgon et al. 2020) [48]
Sarawak, Malaysia	Niah Cave	45–39 ka	1	*P*. *pygmaeus* (Hooijer 1960, Harrison et al. 2014) [6, 8]

Location and chronology of sites and original assignments of fossil *Pongo* specimens used for geometric morphometric analyses.

Our aim is to obtain morphological and molecular data to characterize the diversity in fossil *Pongo* and explore their phylogenetic relationship to modern species. We aim particularly to verify if our results obtained with geometric morphometrics of the EDJ on a set of lower molars and paleoproteomics of dental enamel complement each other and fit with the distinction of extinct species established in previous studies [6]. Furthermore, we aim to characterize enamel protein sequences of extinct species and compare them with modern orangutan protein sequences.

## 2. Material and methods

### 2.1 Materials

Our analysis surveyed *Pongo* samples from continental and insular sites in Southeast Asia. A total of 114 teeth were analyzed in this study, including 71 fossils and 43 teeth from extant species. The details of their location and chronology, along with the original allocations of *Pongo* specimens are shown in Table 1. The fossil sample (Table 1 and S1 Table) comprises 35 teeth from Vietnam, 25 from Indonesia, 9 from China, one from Malaysia and one from Laos, all lower molars. Within the modern molars 20 are allocated to *Pongo abelii* and 23 to *Pongo pygmaeus*, all with known molar positions on the tooth row. For paleoproteomics, fifteen fossil teeth were sampled (Table 2). The sample set, either premolar, molar or canine, permanent or deciduous, includes six teeth from Laos (Tam Hay Marklot n = 5, Tam Hang South n = 1), three from Vietnam (Duoi U’Oi n = 2, Hoà Binh n = 1), four from Malaysia (Niah Cave), one from Indonesia (Sangiran) and one from Thailand (Tham Prakai Phet).

**Table 2:** Fossil specimens for paleoproteomic analyses.

Region	Fossil site	Age	n	Species
Vietnam	Duoi U’Oi	70–60 ka	2	*P*. *pygmaeus* [45, 50]
	Hoà Binh	uncertain	1	*Pongo* sp. [4]
Indonesia	Sangiran Dome	1.5 Ma to 500 ka	1	*Pongo* sp. [61]
Laos	Tam Hay Marklot	38.4–13.5 ka	5	*Pongo* sp. [48]
	Tam Hang South	94–60 ka	1	*P*. *pygmaeus* [49, 50]
Sarawak, Malaysian Borneo	Niah Cave	45–39 ka	4	*P*. *pygmaeus* [6, 8]
Thailand	Tham Prakai Phet	Late Pleistocene	1	*Pongo* sp. [51]

Location and chronology of sites and original assignments of fossil specimens used for paleoproteomic analyses.

### 2.2 Fossil sites

#### 2.2.1 China

The Ganxian Cave located in the Bubing Basin, Guangxi Zhuang Autonomous Region, is dated between 362 ± 78 ka and 168.9 ± 2.4 ka by Uranium–series (U-series) and coupled Electron Spin Resonance (ESR) and /U-series dating [38]. Specimens from this site have been allocated to *Pongo weidenreichi* [38]. The Chinese Apothecary collection comprises isolated teeth of several specimens attributed to *Sinanthropus officinalis* (*Homo erectus)*, *Hemanthropus peii*. *Gigantopithecus* and fossil *Pongo*. The teeth were purchased by Gustav Heinrich Ralph von Koenigswald in traditional Chinese drugstores, and therefore their provenance and dating remain unknown [39, 40].

#### 2.2.2 Vietnam

The Lang Trang cave system is composed of multiple breccia deposits. The fauna has been biochronologically dated to ~80 ka, whereas the ESR dates for the cave breccias gave an age interval of 480–146 ka, which makes this site difficult to characterize chronologically. The molars used in this study have first been assigned to *Pongo pygmaeus* [41–43]. The *Pongo* specimens from two fossil assemblages, Tham Om (province of Nghe An) and Hang Hum (province of Yên Bai) in northern Vietnam, are supposed to be 300–200 ka and 140–80 ka respectively, based on biochronology [3, 44]. The Duoi U’Oi site located in the Hoà Binh province is dated to 70–60 ka by Optically stimulated Luminescence (OSL) and U-series dating and teeth have been identified as *Pongo pygmaeus* [45]. No dates are reported for the *Pongo* specimen from Hoà Binh, since radiocarbon dating was inconclusive [4]. The Mai Da Dieu Rockshelter is located in the limestone mountains of Thanh Hoa Province. *Pongo* fossils derived from layer 1 with radiocarbon dates of 30–24 ka [46]. The Thung Lang specimens [47] have never been dated.

#### 2.2.3 Laos

The specimens from Laos come from Tam Hay Marklot Cave, in which a set of mammal teeth have been dated to 38.4–13.5 ka by Uranium-thorium (U-Th) [48] and from Tam Hang South dated to 94–60 ka using OSL and U-series methods on fossiliferous deposits [49, 50]. Both sites are located in the Huà Pan province in northeastern Laos. Molars are identified as *Pongo pygmaeus*.

#### 2.2.4 Thailand

Tham Prakai Phet cave is located in northeast Thailand and the occurrence of fossil *Pongo* is biochronologically estimated to the Late Pleistocene. Specimens are attributed to *Pongo* sp. [51].

#### 2.2.5 Malaysia

The Late Pleistocene human occupation at Niah Cave, Sarawak (Malaysian Borneo) is dated back to 50–45 ka by radiocarbon dating of charcoal and U-series analyses on bone. Specimens associated with it are allocated to *Pongo pygmaeus* [8–10].

#### 2.2.6 Indonesia

The specimens from Indonesia come from the Early to Middle Pleistocene Sangiran Dome, Java. The hominid bearing layers at the Sangiran Dome cover an age range of 1.5 Ma to 500 ka by ^40^Ar/^39^Ar, fission-track and Uranium-lead (U–Pb) dating [52, 53]. The exact provenance of the *Pongo* material from Sangiran is not documented, hence it is not possible to attach a secure date [54, 55]. The Punung specimens studied here were collected by Gustav Heinrich Ralph von Koenigswald in the early 1930s in the Punung I and II localities [56]. The Late Pleistocene Punung fauna represents a tropical rainforest fauna for the first time on Java and is dated to 128–117 ka using luminescence and U-series methods on sediments from the nearby Gunung Dawung site, named Punung III [57]. Fossils from Punung are allocated to *Pongo pygmaeus* [58]. Drawhorn [11] suggested to assign these specimens to *Pongo javensis*, which was echoed by Harrison et al. [6]. However, *Pongo javensis* as a new species was never formally published. The site, located in the Gunung Sewu karst region in East Java, has been recently reanalyzed with a new interpretation of fossiliferous deposits [59].

The specimens from Indonesia and the Chinese Apothecary Collection are housed at the Senckenberg Research Institute and Museum Frankfurt am Main, Germany. *Pongo* molars from several Vietnamese Pleistocene sites are all housed at the Institute of Archaeology in Hanoi, Vietnam. The new faunal assemblages from Laos are curated at the National Museum in Vientiane, Laos and the National Natural History Museum in Paris, France for the old collection of Thung Lang. Fossil specimens from Ganxian Cave are curated at the Natural History Museum and Anthropology Museum of Guangxi, China. Fossils from Niah Cave were housed at the University of Cambridge, UK, when they were studied but have since been returned to Sarawak Museum, Malaysia. No permits were required for the described study, which complied with all relevant regulations.

### 2.3 Methods

#### 2.3.1 Morphological analyses

*X-ray microcomputed tomography (X-μCT)*. All specimens were scanned using the X-ray microfocus sources (X-μCT) (Phoenix Nanotom S 180; General Electric Company) with the following parameters: 100–160 kV, 0.11–90 μA, 0.14–0.36° between each projection and a voxel size ranging between 0.02 and 0.04 mm.

*Segmentation of microCT data*. Segmentation and landmarking were performed in Avizo v.7.0 (FEI Visualization Sciences Group). Dentine surfaces of each specimen were segmented using the magic wand tool with manual corrections locally where contrast was low. In cases where resolution and contrast were too weak, the interpolation tool was used. Interpolations were also utilized for reconstructions of morphological structures such as dentine horn tips in cases of damage due to taphonomy or occlusal wear (see Zanolli et al. [63]). Volumetric models were generated for each specimen.

*Molar position assessment*. As noted by Hooijer [1], Badoux [56], Groesbeek [64] and Harrison [65], and confirmed by our observations, it was not possible to reliably determine the serial position of the isolated molars of our fossil samples. Nevertheless, we tried to identify M3s among the isolated fossil teeth to minimize any potential bias in the phylogenetic analyses as M3s show typical traits such as a marked distal tapering and low cusps and dentine horns, as well as the absence of an interproximal distal contact facet. However, the latter feature is not necessary always reliable as there can be the presence of a M4 (with a frequency of 7–13% in extant orangutans [56, 66], and in case of unerupted or just erupted M1 or M2, since the distally positioned teeth are not erupted, there is no interproximal distal contact facet. Based on majority consensus among three observers (JK, CZ, AMB) molar positions were assessed (S3 Fig). Analyses with all molar positions combined in one single CVA analyses are shown in S4 Fig to compare how this affects the taxonomic signal.

*Geometric morphometric analyses*. We mirrored all left molars in the sample, so that all teeth are virtually considered as right antimeres to ensure that (semi)landmarking of each specimen is homologous. We placed landmarks and semilandmarks in the same order as follows: Protoconid—Metaconid—Entoconid—Hypoconulid—Hypoconid—Protoconid. Five landmarks were placed on the surface of the tip of the main dentine horns combined with 142 semilandmarks along the marginal ridges connecting the dentine horns of the enamel-dentine junction (EDJ) using the Bspline tool. Generalized Procrustes analyses, principal component analyses (PCA) and canonical variate analyses (CVA) based on Procrustes shape coordinates and geographical origin as groups were carried out using the software R version 4.1.1 [67]. Groups were defined as China, Vietnam and Indonesia for the fossil specimens, and Sumatran orangutans (*Pongo abelii*) and Bornean orangutan (*Pongo pygmaeus*) for the living species. Considering the ambiguity of metameric position for a number of specimens, we conducted geometric morphometric analyses using various (sub)sets of specimens that include: (i) M1 and M2 for fossil *Pongo* based on our molar position classification and M1 and M2 of modern *Pongo*, (ii) M3s for fossil *Pongo* based on our molar position classification (including specimens for which the M3 position is ambiguous and could represent M2s) and M3s of modern *Pongo*, and (iii) all molar positions available for fossil and modern *Pongo*. Since CVA computation requires the number of variables to be much smaller than the number of specimens, we computed the CVA based on a subset of the first PC scores (12 PCs for the M1–M2 analysis, and 15 PCs for the M3 analysis) showing the highest degree of correct classification (screening the correct classification results and selecting the minimum number of PC scores enabling to reach the optimum of correct classification) [68]. This choice of the PC scores subset is a compromise between including a sufficient proportion of overall shape variation and limiting the number of variables to avoid unrealistic and unstable levels of discrimination.

Specimens from Sangiran and those used for paleoproteomic analyses were excluded from the CVA and projected a posteriori in the plots because the number of these specimens per locality is limited and computing a CVA with less than 5 specimens would not provide reliable results. Cross-validations were run to check that CVA results do not show spurious group discrimination by looking at the cross-validated classification accuracy (S2 and S3 Tables). Cross-validated CVA plots were inspected as well to compare if there is broad agreement on group distribution in the morphospace (S1 Fig). We tested allometry based on the Pearson correlation coefficient between the centroid size and CV1, CV2 and CV3, respectively. In addition, we calculated the squared correlation coefficient between centroid size and canonical variates.

#### 2.3.2 Paleoproteomics

*Protein extraction*. Ancient protein extraction was carried out in clean laboratory facilities at the University of Copenhagen dedicated to the extraction of ancient biomolecules. A sterilized drill was used to remove flakes of enamel from intact molars and premolars. One specimen (SMF 8864) was embedded in resin for enamel histological analyses in a previous study [69] prior to laboratory analysis. This specimen (SMF 8864) was coated with two different resins, Crystalbond 509 (SPI Supplies) soluble in acetone which directly covered the teeth and a second layer coating with epoxy resin (EpoThin 2; Buehler), which is not soluble [69]. The specimen embedded in resin was cut and submerged in acetone for 3 hours to remove the resin. The specimen was washed with distilled water and subsequently with 10% trifluroacetic acid (TFA) for 2 minutes to remove residues and left for drying for 12 hours.

For each specimen, approximately 25 mg of enamel was sampled from the non-occlusal portion of the crown. Care was taken to remove any residual dentine remaining on enamel flakes following drilling. These enamel flakes were subsequently crushed to a rough powder, which was demineralized using 10% TFA at 4°C for 24 hours. Samples were subsequently processed using a digestion-free protocol developed for recovering degraded enamel proteins [36]. C-18 StageTips [70] were used to collect and desalt peptides. Negative extraction blanks were processed alongside each batch of samples, to control for contamination.

*LC-MS/MS*. StageTips were eluted into a 96-well MS plate using 30 μL of 40% acetonitrile (ACN) 0.1% formic acid (FA). To remove the ACN and concentrate the samples, samples were vacuum-centrifuged until approximately 3 μL of sample remained. Finally, samples were resuspended in 10 μL of 5% ACN 0.1% TFA, except for SMF 8864, which was resuspended in 6 μL instead based on observations of peptide concentrations at this site from previous tested suid specimens (not reported). Liquid chromatography-tandem mass spectrometry (LC-MS/MS) was used to analyse the samples, using established protocols for paleoproteomic samples [36, 71]. Samples separation was completed on a 15 cm column (75 μm inner diameter) in-house laser pulled and packed with 1.9 μm C18 beads (Dr. Maisch, Germany) on an EASY-nLC 1200 (Proxeon, Odense, Denmark) connected to an Exploris 480 Orbitrap mass spectrometer (Thermo Scientific, Bremen, Germany). 0.5 μL was injected for all samples except SMF 8864, for which 3 μL was used, based on the total ion current of small test injections (0.5 μL injections over 15 min). Buffer A was milliQ water and the peptides were separated with increasing buffer B (80% ACN and 0.1% FA) with a 77 min gradient, increasing from 5% to 30% in 50 min, 30% to 45% in 10 min, 45% to 80% in 2 min, held at 80% for 5 min and decreased back to 5% in 5 min and held for 5 min. Flow rate was 250 nL/min. An integrated column oven was used to maintain the temperature at 40°C. A wash blank, using 5% ACN 0.1% TFA, was run in between each sample to hinder cross-contamination.

The Exploris 480 recorded full scan mass spectra (MS1) at a resolution of 120,000 over the m/z range 350–1400 with a target AGC of 300% and a maximum injection time of 25 ms. Spray voltage was set to 2kV, the heated capillary at 275°C, and the S-lens RF level was at 40%. HCD-generated product ions (MS2) were recorded in data-dependent top 10 mode with a maximum ion injection time set to 118 ms and a target ACG value of 200%, recorded at a resolution of 60,000. HCD collision energy was set at 30% and the isolation window was 1.2 m/z with the dynamic exclusion set to 20 s.

*Data analysis*. For an initial database search, a broad taxonomic database was constructed by translating those proteins commonly encountered in enamel from publicly-available genomic data for catarrhines [33, 37]. These sequences were supplemented with sequences from various other common bone and dentine proteins available on Uniprot for *P*. *abelii* and *P*. *pygmæus*. A second reference database on common laboratory contaminants was also included in each database.

Proprietary.raw files generated by the mass spectrometer were searched using MaxQuant (v1.6.3.4) [72], for the main protein sequence reconstruction. Raw files were additionally searched using PEAKS, via the spider algorithm [73], and PFind, via its open search strategy to potentially discover single amino acid polymorphisms (SAPs) [74]. Initial database searches with MaxQuant were run against the two aforementioned databases. A minimum Andromeda score of 60 was set for both modified and unmodified peptides, with a minimum length of seven amino acids and a maximum of 25 amino acids. The default tolerance settings were used– 20 ppm for the first search and 4.5 ppm for the final search, with a fragment mass tolerance of 20 ppm. The peptide false-discovery rate (FDR) was set to 1.0%. Delta score was set at zero for both modified and unmodified peptides. No fixed post-translational modifications (PTMs) were set. The following variable modifications were included: glutamine and asparagine deamidation, oxidation of methionine, proline and tryptophan, phosphorylation of serine and threonine, and pyroglutamic acid from N-terminal glutamic and aspartic acid.

Similar search parameters were selected in PFind and PEAKS. In PFind, the peptide FDR remained 1%, but the protein FDR was set to 10%, and the peptide mass window was set at 350–5000. In PEAKS, the average local confidence was set to 90%. Potential SAPs discovered in PFind and PEAKS were then included in manually-constructed alternative protein sequences, to be included in a final validatory run using MaxQuant alongside the initial hominid-only database. Besides the updated database, the final MaxQuant run used the same parameters as the initial database searches, but also included an additional PTM (ornithine conversion from arginine).

Common laboratory contaminants and reverse hits from the FDR calculation were removed from consideration for all further aspects of the analysis. Peptide sequences matching to spectra with poorly-covered ion series were discarded prior to sequence reconstruction. Protein sequences were reconstructed using MAFFT v.7 [75], specifically the–addfragments option, for which individual peptide sequences were aligned to the *Pongo abelii* sequences for each protein, prior to generating a consensus sequence. Deamidation was calculated using a python script on the evidence.txt output file from the final MaxQuant run, as described in Mackie et al. [72] and Cappellini et al. [36]. Figures were constructed using GGplot2 [76].

*Protein reference dataset for phylogenetic analyses*. We created a reference dataset for 8 proteins common in dental enamel: alpha-2-HS-glycoprotein (AHSG), albumin (ALB), ameloblastin (AMBN), amelogenin (AMEL), amelotin (AMTN), collagen alpha-1(XVII) (COL17A1), enamelin (ENAM), and matrix metalloproteinase-20 (MMP20), that includes all extant hominid genera. Additionally, the proteins collagen alpha-1 (I) (COL1A1) and collagen alpha-2 (I) (COL1A2), which are common in dentine, were included in the same dataset in case traces of dentine were accidentally co-extracted.

Since protein data for *Pongo* and other non-human great apes is scarce, we translated the proteins of interest from publicly available genomic data [17, 77–80]. This translated dataset includes 27 *Pongo* individuals from all 3 extant species of the *Pongo* genus (15 *P*. *pygmaeus*, 11 *P*. *abelii*, 1 *P*. *tapanuliensis*) and captures much of the within-genus protein diversity of these 9 proteins. We combined this reference dataset with the protein sequences from the 15 fossil *Pongo* samples.

*Protein phylogenetic analysis*. We created a multiple sequence alignment (MSA) for each one of these 9 proteins using MAFFT [70]. We then identified all positions in the alignment, covered by at least one fossil sample, with either an isoleucine (I) or leucine (L) amino acid. These two amino acids are isobaric and are impossible to differentiate using the mass spectrometric procedure described here [33]. For those positions, if all modern samples had one of the two amino acids, we parsimoniously switched the fossil samples to the same. If both amino acids are present within the modern individuals, we switched all samples of the alignment, both modern and fossil, to a leucine. Finally, we concatenated the 9 MSAs into a single alignment and used it as input for two phylogenetic software: PhyML (Maximum likelihood) [81] and MrBayes (Bayesian) [82]. Using these two software tools, we generated two species-phylogenetic trees.

## 3. Results

### 3.1 Geometric morphometrics

#### 3.1.1 Extant *Pongo*

**Fig 1:**
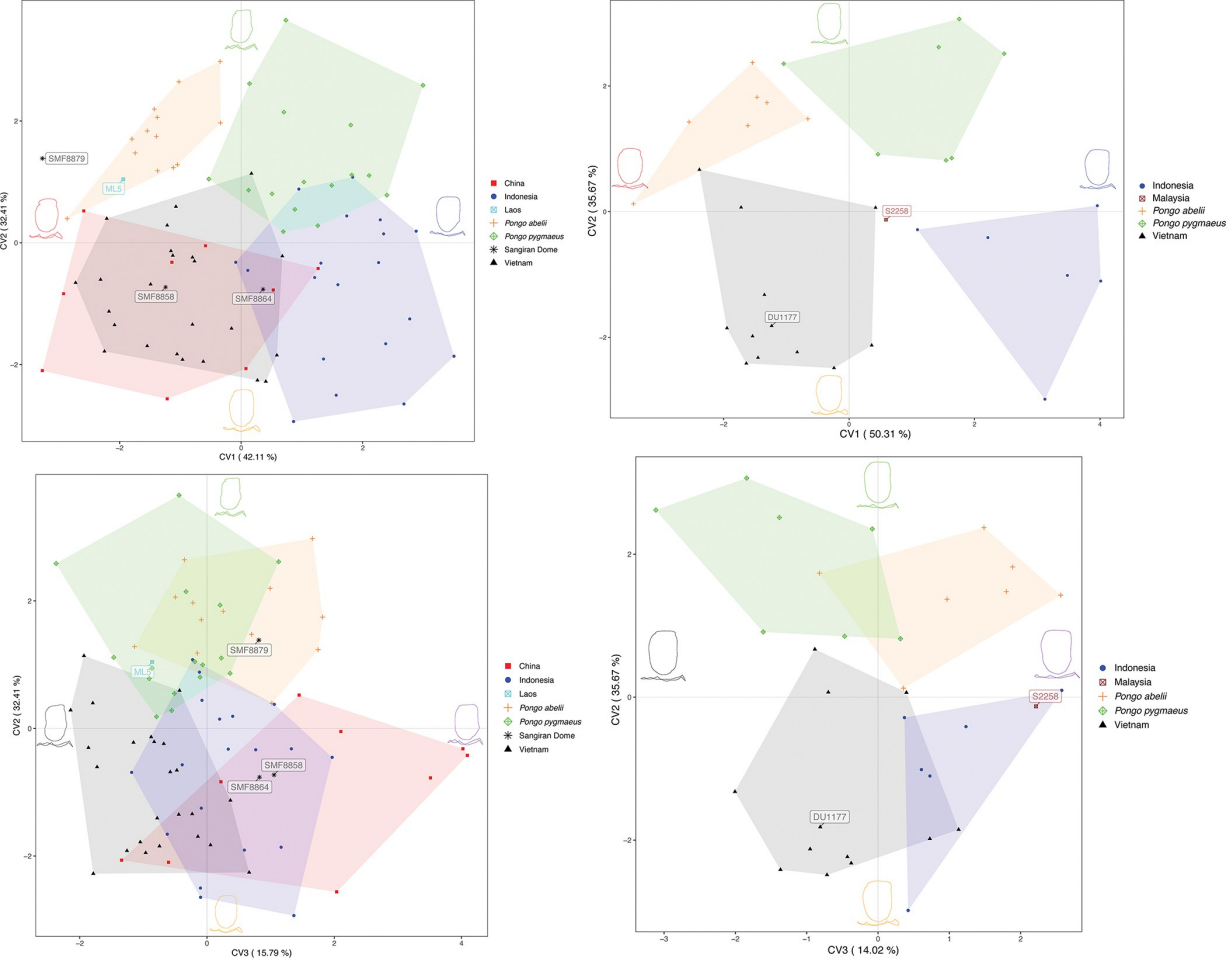
**a.** Canonical variate analyses (CVA) of the enamel-dentine-junction (EDJ) based on M1s and M2s. Specimens from the Sangiran Dome (Java, Indonesia) and Laos are projected a posteriori to the analyses due to the limited number of available specimens for these sites. All these specimens are highlighted with respective labels (SMF8864, SMF8858 and SMF8879, Sangiran Dome, Java; ML5, Tam Hay Marklot, Laos). EDJ shape changes and outline of crowns along canonical variates are illustrated. The maximum of extreme shape change along CV1 is illustrated in blue, while the minimum is shown in red, for CV2: maximum = green, minimum = orange; for CV3 maximum = purple, minimum = black. Specimens SMF 8864 (Sangiran Dome), and ML5 (Tam Hay Marklot, Laos) are included in the paleoproteomic analyses as well. **1b.** CVA of the EDJ based on M3s. One specimen from Malaysia is projected a posteriori to the analyses due to the limited number of available specimens for this site and highlighted with the respective label (S2258; Niah Cave, Malaysian Borneo). EDJ shape changes and outline of crowns along canonical variates are illustrated. The maximum of extreme shape change along CV1 is illustrated in blue, while the minimum is shown in red, for CV2: maximum = green, minimum = orange; for CV3 maximum = purple, minimum = black. Specimens DU1177 (Duoi U’Oi, Vietnam) and S2258 (Niah Cave, Malaysian Borneo) are included in the paleoproteomic analyses as well.

*CVA analyses with M1 and M2 combined*. In the canonical variate analysis (CVA) (Fig 1A), CV1 (axis X) and CV2 (axis Y) represent 42.11% and 32.41% of the total variance, respectively. The CVA plot shows that most of the *P*. *pygmaeus* molars are grouping on the positive part of CV1, whereas the *P*. *abelii* molars appear clearly distinct on the negative part. Along this axis, *P*. *pygmaeus* molars group together with Indonesian fossil molars, whereas the majority of fossils from Vietnam and China cluster on the negative part together with *P*. *abelii*. The shape change along CV1 indicates higher and more inward curved dentine horns for Vietnamese and Chinese specimens and *P*. *abelii*. *P*. *pygmaeus* and Indonesian molars show lower dentine horns, especially less protuberant hypoconid.

A more evident separation of extant and fossil specimens occurs along CV2 where extant species cluster in the positive part whereas fossil groups are in the negative area. However, some little overlaps exist between fossils from Vietnam and Indonesia and modern molars, especially *P*. *pygmaeus*, while Chinese molars range show no overlap with ranges of modern taxa.

Along CV2, the outline of the tooth changes. It shifts from an elongated, almost rectangular, and narrow crown mesiodistally expressed through buccal and lingual dentine horns. Dentine horns are narrower and more closely arranged in modern *Pongo* molars compared to the more peripheral dentine horns in the fossil molars with a rounder and shorter enamel-dentine junction (EDJ) outline. The shape change along CV1 is mainly driven by the height of the dentine horns, while CV2 is mainly determined by the position of the dentine horns and by the shape of the outline.

The degree of cross-validated correct classification (reported in S2 and S3 Tables, illustrated in S1 Fig) for *P*. *abelii* and *P*. *pygmaeus* is 77% and 69%, respectively, which shows that the two species of living *Pongo* included in this study are relatively well distinguished from each other and from fossil groups. The overall classification accuracy is 67% indicating a general separation of groups, even if some overlap exists. There is no significant allometric trend between fossil and modern specimens regarding the grouping of molars along CV1 and CV3 (*p* = 0.89 and *p* = 0.44, respectively). Along with CV2 there is little allometry with a p-value of < 0.05 and r^2^ = 0.22.

#### 3.1.2 Fossil *Pongo*

Fossils from the Middle to Late Pleistocene sites in Vietnam share the same morphospace along all 3 CVs and cluster on the negative area (Fig 1A). The cross-validated classification results show that 63% of Vietnamese fossils were correctly classified with substantial overlap to Chinese and Indonesian fossils. The overlap especially concerning China and Vietnam is evident in CV1 vs. CV2 (Fig 1A) and in the cross-validated classification results, which show that 44% of Chinese fossils were correctly classified, whereas 33% were identified as Vietnamese fossils (S2 Table). In CV2 vs. CV3 (Fig 1A; CV3 representing 15.79% of the total variance) fossils from China separate to some extent from Vietnam indicating some shape differences captured along CV3. Moreover, the Chinese sample is showing a high variation along CV3 due to the large morphospace occupied, which might indicate high variability of the Chinese group (Fig 1A). The majority of the specimens group on the far positive part along CV3, which is characterized by slightly sharper dentine horns with a prominent entoconid as opposed to the far negative area along CV3 where most of the Vietnamese fossils group, which show a relatively low entoconid expression. Three specimens from Ganxian (GX010, GXI007, GX3012) partially overlap with some specimens from Vietnam. The rest of the Middle Pleistocene Ganxian and Chinese Apothecary specimens fall together in the positive space of CV3. The Punung specimens overlap with fossil *Pongo* from Vietnam and China. Several specimens from Punung fall within the range of *P*. *pygmaeus* as well. Indonesian fossils show 74% of correct classification.

The molars from Sangiran (Indonesia), Niah Cave (Malaysia) and Tam Hay Marklot (Laos) were projected a posteriori to the CVA plot due to the limited number of specimens. One molar from Laos labeled “ML5” in Fig 1A falls close to the *P*. *abelii* group, a result supported by the typicality probability (Table 3). Specimen SMF 8858 from the Sangiran Dome groups with fossils from China, and Vietnam, whereas SMF 8864 falls in the morphospace shared by fossils from Punung, Indonesia and China. Both Indonesian specimens are well discriminated from the extant species. Typicality probabilities show a higher affinity of SMF 8858 to the Chinese group, and of SMF 8864 to the Indonesian group (Table 3). Specimen SMF 8879 does not show particular affinity to any of the fossil groups, but exhibits a close proximity to *P*. *abelii*, which is also suggested by the typicality probabilities.

**Table 3:** Typicality probabilities of the investigated specimens.

Specimen ID	China	Indonesia	*Pongo abelii*	*Pongo pygmaeus*	Vietnam
ML5 (Marklot, Laos)	0.02	0.00	**0.54**	0.03	**0.33**
SMF 8858 (Sangiran Dome, Indonesia)	**0.79**	**0.05**	**0.14**	0.01	**0.43**
SMF 8864 (Sangiran Dome, Indonesia)	**0.39**	**0.67**	**0.05**	**0.11**	**0.31**
SMF 8879 (Sangiran Dome, Indonesia)	0.01	0.00	**0.40**	0.00	0.01
S2258 (Niah Cave, Malaysian Borneo)	-	0.00	0.00	0.00	**0.97**

Significant probabilities (above statistical threshold of 0.05) are highlighted for each fossil specimen.

*CVA analyses of M3*. The CVA analyses for M3s follow the trend seen in M1 and M2s regarding the grouping of extant and fossil specimens. However, Chinese fossils are missing because of the lack of identifiable M3s in the sample (Fig 1B; CV1 representing 50.31% of the total variance and CV2 representing 35.67%). *P*. *abelii* and Vietnamese fossils cluster in the negative area along CV1 with Indonesian fossils and *P*. *pygmaeus* grouping on the positive part. These results are similar to the previous plots where M1 and M2 are analyzed. The shape change along CV1 exhibits more inward curved dentine horns with slightly more prominent distal dentine horns for specimens from Vietnam and *P*. *abelii* than for specimens from Indonesia and *P*. *pygmaeus*. The shape change along CV2 shows a more rectangular and elongated EDJ with more closely placed dentine horns for modern molars, whereas fossil specimens exhibit a shorter and rounder EDJ outline. There is no significant allometric trend between fossil and modern specimens regarding the grouping of molars along CV1 and CV2 (*p* = 0.5 and *p* = 0.17, respectively).

The degree of cross-validated correct classification (reported in S4 and S5 Tables, illustrated in in S1 Fig) for *Pongo abelii* and *Pongo pygmaeus* is 43% and 43%, respectively. Two species of living *Pongo* included in this study are somehow distinguished from each other and from fossil groups but the cross-validated classification results for M1–M2 showed higher percentages. The overall classification accuracy is 50% indicating substantial overlap, however fossils from Punung were well separated from all other groups with 83%. The specimen from Niah Cave (Malaysia) labeled “S2258” projected a posteriori (Fig 1B) clusters close to Vietnamese specimens along CV1 and Indonesian specimens along CV3. The typicality probability for this specimen shows the highest affinity to Vietnamese fossils (S2 Table).

### 3.2 Paleoproteomics

**Fig 2:**
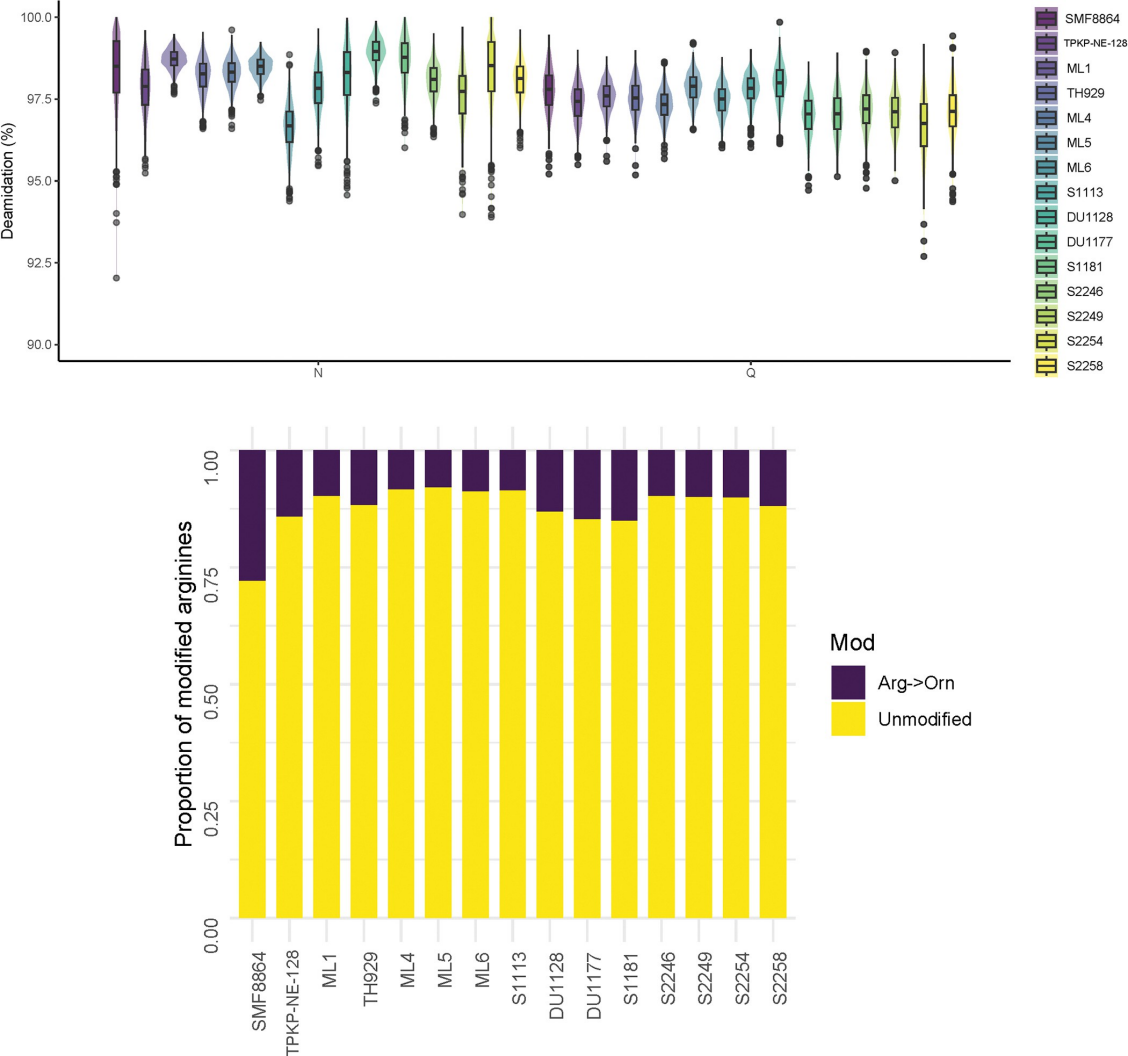
**(a)** Deamidation rates for each fossil specimen. Note range of values (80–100) (see S6 Table for data). **(b)** Arginine to ornithine ratio for each fossil specimen. Proportion of arginines that have been modified in the form of conversion to ornithine, by sample (see S6 Table for data).

**Fig 3:**
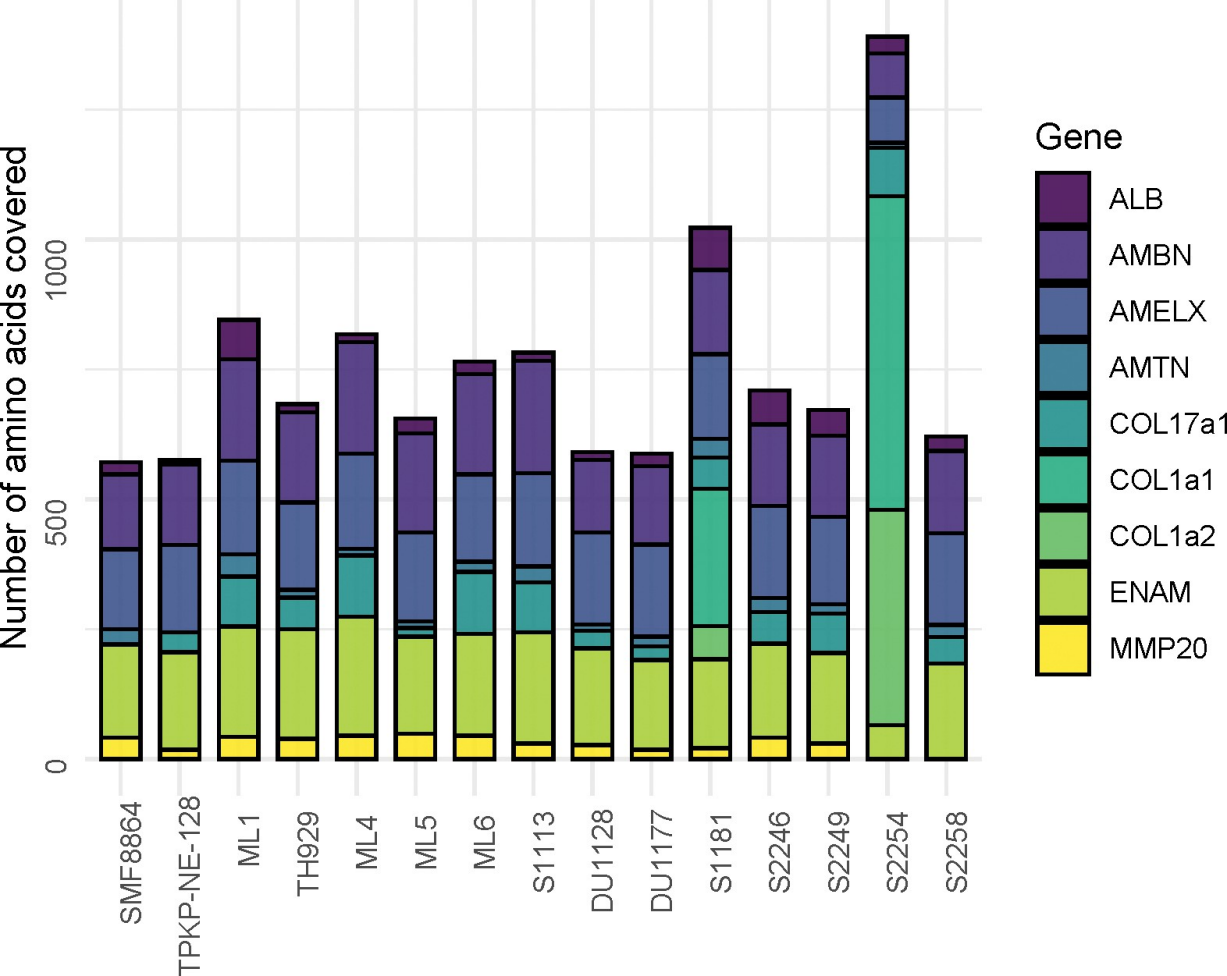
Amino acid coverage per protein. Bar plot displaying number of amino acids covered by protein for each specimen.

The high likelihood of authenticity of proteins retrieved from fossil samples is suggested by the high rates of deamidation, which are similar across all specimens in the analysis (Fig 2A and S6 Table). Moderate rates of arginine to ornithine conversion are also observed in the ancient samples (Fig 2B), though a noticeably higher rate is observed in the Middle Pleistocene Sangiran specimen SMF 8864, reflecting its greater antiquity. In spite of the increased level of degradation observed in its paleoproteomic profile, SMF 8864 preserves a suite of enamel proteins comparable to those of the other Late Pleistocene specimens, and an amino acid coverage approaching the median of the Late Pleistocene specimens (Fig 3). In addition to the typical suite of enamel proteins (AMBN, AMELX, AMTN, ENAM, MMP20) and serum albumin (ALB), a high abundance of collagen peptides (COL1A1 and COL1A2) is observed in a pair of samples (S1181and S2254). The presence of COL1A1 and COL1A2 is likely due to traces accidentally co-extracted from dentine in these samples (Fig 3).

#### 3.2.1 Sequence variation

**Fig 4:**
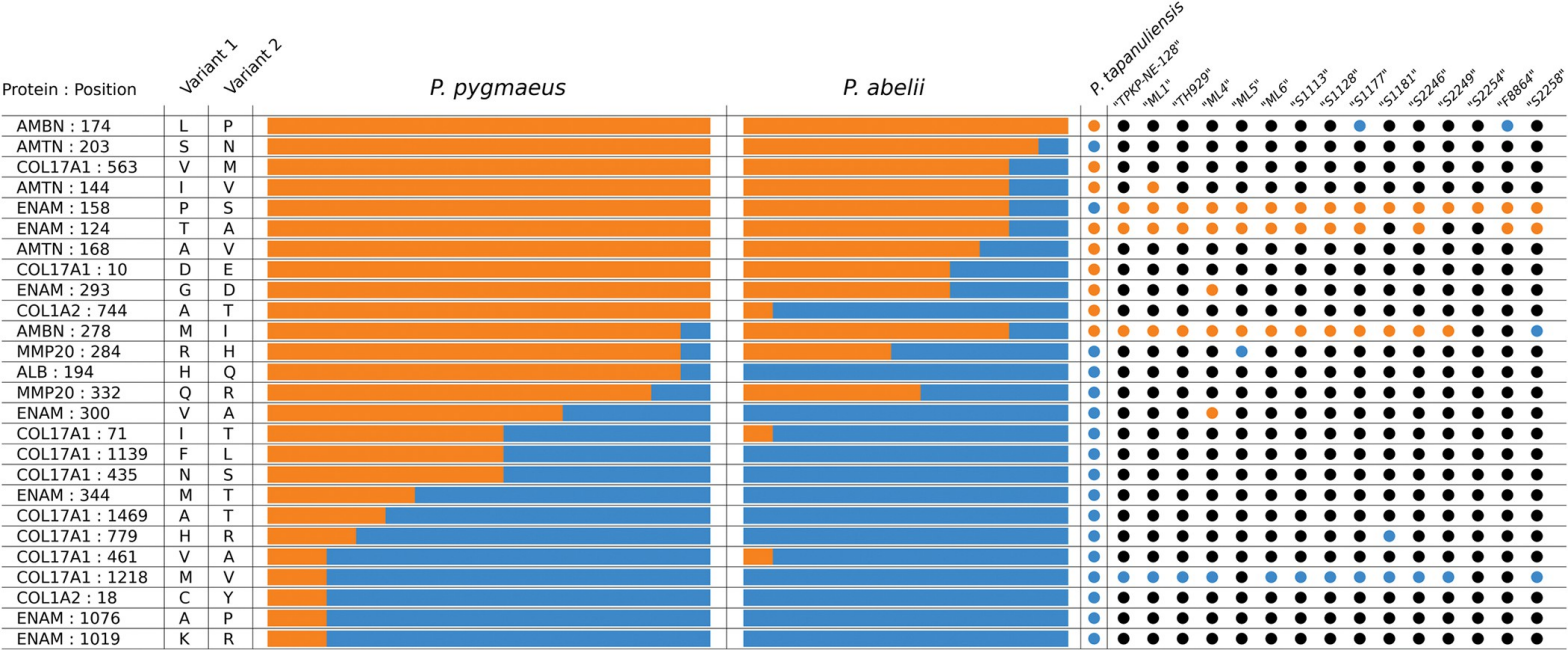
Amino acid sequence variation within Pongo. The first column represents the position on the respective protein, where 2 alternative variants can appear in the genus (a single amino acid polymorphic site). These variants appear in different frequencies across extant *Pongo* species and fossil *Pongo* specimens (variant 1 = orange, variant 2 = blue, if no position is covered = black).

After inspecting the alignments of the *Pongo* clade, we were able to identify few single amino acid polymorphisms (SAPs) within that clade (Fig 4). Although no SAP is exclusive to any species of *Pongo*, some of them appear with different frequency within each species. We were able to identify a single SAP in AMBN (Position 174 in the *Pongo abelii* canonical Ensembl transcript of AMBN—ENSPPYT00000017210.2) present only in the fossil samples and all the other non-*Pongo* hominids (Fig 4). This position was covered in only 2 out of the 15 ancient samples we analyzed, with one originating from the Early to Middle Pleistocene Sangiran site (Java) and the other from the Late Pleistocene site of Duoi U’Oi (Vietnam). This SAP should be interpreted cautiously due to its restricted occurrence and its limited value on broader evolutionary inferences.

#### 3.2.2 Protein phylogenetic results

**Fig 5:**
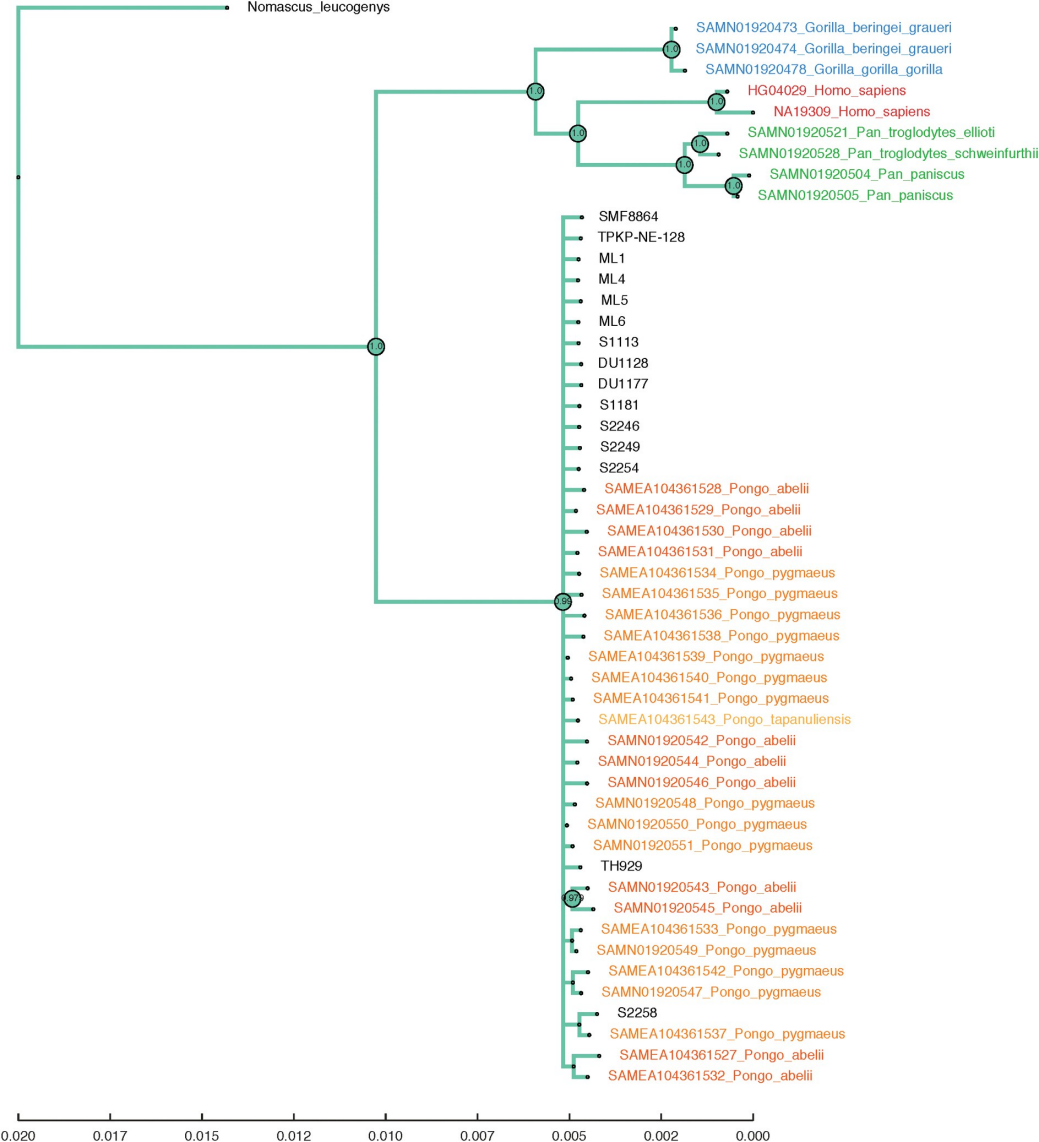
Phylogenetic tree. Bayesian phylogenetic tree created by using MrBayes based on dental protein sequences of fossil *Pongo* samples and several hominid species (*Gorilla gorilla*, *Gorilla beringei graueri*, *Pan paniscus*, *Pan troglodytes schweinfurthii*, *Pan troglodytes ellioti*, *Pongo pygmaeus*, *Pongo abelii*, *Pongo tapanuliensis* and *Homo sapiens*). *Nomascus leucogenys* was added as an outgroup. Both modern and fossil *Pongo* samples are grouped into a single clade with high confidence and without any substructure.

Both the Bayesian tree (Fig 5) from MrBayes and the maximum likelihood tree (S2 Fig) from PhyML capture the established phylogeny of the four extant hominid genera, chimpanzees (*Pan)*, gorillas (*Gorilla)*, orangutans (*Pongo)* and humans (*Homo*) with gibbons (*Nomascus*) as an outgroup. In the Bayesian tree, all the fossil *Pongo* samples are placed in the same clade as all modern *Pongo* samples with high support, whereas for the Maximum Likelihood tree this placement has moderate support. In both trees there is no statistical support for any substructure within the *Pongo* genus, including no separation of the modern species (*P*. *pygmaeus*, *P*. *abelii*, *P*. *tapanuliensis*) or the modern samples from the fossil ones.

## 4. Discussion

### 4.1 Does the analysis of prominent features of the EDJ confirm species concepts in the genus *Pongo*?

#### 4.1.1 Molar position assessment

Previous studies of external size and morphology of molars demonstrated that metameric variation in *Pongo* is too high to accurately determine the serial position of molars among isolated teeth [1, 56, 64, 65]. Our inter-observer test to identify molar position confirmed these previous results, which led us to run geometric morphometric analyses using some subsets of molar positions (M1–M2 on the one hand, and M3 on the other), as well as with all molar positions combined. Our results show that, whether the molars are tentatively discriminated by position or combined together, there is a broad agreement among grouping of extant and fossil groups and similar proximities between each other in the morphospace (S4 and S5 Figs). The overlap between groups is however considerably higher when all molar positions are combined, which leads to a less distinct taxonomic signal, but the statistical power is higher since there are more specimens, and the risk of tooth misattribution is avoided.

However, running geometric morphometric analyses separately for each molar position can also lead to biased results if molar positions are not correctly identified for isolated fossil specimens. Our molar type assessment revealed a tendency to assign uncertain cases to the M2 position. As a result, M2 assignments were overrepresented in our dataset. Furthermore, in several cases there was no consensus among all three observers and M3 were assigned much less frequently than the other positions (see S3 Fig for the results of the molar type assignments). Although the absence of interproximal distal contact facet is generally a diagnostic feature of M3s, it can be absent on M1 if M2 is not yet emerged, likewise on M2 if M3 is not emerged. There is also the possibility of the presence of a M4 in *Pongo*, which is relatively high, around 7–13% in extant species [56, 66] and similarly high in extinct *Pongo* [1]. Accordingly, a M3 would have a distal wear facet when an M4 is present. Our results show that, even when mixing molar positions, the different groups represented by extant and fossil specimens show broad agreement across all CVA plots suggesting morphometric distinction of extant *Pongo* species from fossil groups, despite the uncertainty of molar positions.

#### 4.1.2 Extant *Pongo*

The large spatio-temporal distribution and marked dental morphological diversity of fossil *Pongo* were linked by numerous scholars to the variability of extant species. Hooijer [1] described a high diversity in extant *Pongo* dentition and argued that fossil teeth from Early to Middle Pleistocene southern China and Late Pleistocene Sumatra fall within the morphological range of *P*. *pygmaeus*, hence allocating these fossils to subspecies, *P*. *p*. *weidenreichi* and *P*. *p*. *palaeosumatrensis*, respectively. Kahlke [2] questioned the validity of both subspecies due to the lack of “clearly defined morphological and metrical differences” and “inadequate characterization” especially of *P*. *p*. *palaeosumatrensis*. Ho et al. [83] suggested merging both fossil subspecies to *P*. *p*. *weidenreichi*. Based on morphology, even *P*. *abelii* was classified as a subspecies of *P*. *pygmaeus* [13, 15]. Later, genetic evidence revealed the molecular distinction of Bornean and Sumatran orangutans, resulting in the elevation of *P*. *abelii* to a full species [84]. Despite the genetic separation of extant *Pongo* species, the outer crown morphology of *P*. *abelii* and *P*. *pygmaeus* shows very little differences, hence isolated teeth of both species mixed together were reported to be impossible to separate [6, 85]. Identifying species of *Pongo* and determining their phylogenetic relationship solely based on the external morphology of isolated dental material remain unsatisfactory due to the large overlap and similarities of dental traits between extinct and extant species [6, 7].

According to our results, the morphometric analysis of the EDJ of *P*. *abelii* and *P*. *pygmaeus* shows distinction both between species and, to some extent, to fossil molars from different geographic origins as well (Indochinese and Sundaic areas). However, the fossil sample set comprises more specimens than our modern groups. Many of the wild orangutan specimens in museum collections were obtained a century ago, thus location and *Pongo* species identification (particularly the identification of *P*. *abelii* from *P*. *tapanuliensis* within Sumatran orangutans) are not always certain. A bigger sample size is needed to adequately capture the differences between *P*. *abelii*, *P*. *pygmaeus* and the recently described *Pongo tapanuliensis* [17]. Capturing the morphospace of extant species would greatly enhance the characterization of fossil molars as well. Despite the constraint due to the sample size, there is a general trend in the distribution of fossil groups relative to modern groups, which will be further discussed.

#### 4.1.3 Fossil *Pongo* from insular Southeast Asia

Based on morphometric traits, our results show that some fossils from Punung, Indonesia, overlap partially with *P*. *pygmaeus* but not with the *P*. *abelii* sample (Fig 1A), although there is overlap with both modern species when considering CV3 (Fig 1B). Some molars from Punung might derive from deposits younger than those of the Late Pleistocene (128–117 ka; [57]) intruding the stratigraphic fossiliferous layers, as recently demonstrated by Kaifu et al. [59]. These teeth seem to be morphometrically very similar to modern species as previously suggested by Badoux [56].

In our analysis, the rest of the Punung material groups in part with fossils from Vietnam and China (Fig 1A). Even though the historical Punung collection was previously considered to be morphologically and metrically identical to *Pongo pygmaeus* [56], a further examination of dental traits established morphometric differences to modern species [6, 11]. Indeed, the relatively smaller size of the Punung dentition compared to that of Late Pleistocene taxa from Sumatra, Borneo, and mainland Southeast Asia led some authors to propose an allocation to a distinct species *P*. *javensis* [6, 11]. Although this new taxon *P*. *javensis* was suggested for the *Pongo* remains from Punung by Drawhorn [11], it was never formally published following the rules of ICZN [86]. Our analysis shows very little allometric influences based on the shape differences of the EDJ morphology along CV2, but only for M1 and M2. The EDJ morphology of Punung fossils shows some distinction to fossils from continental Southeast Asia.

Due to these various overlaps with modern and fossil groups, it is difficult to determine the evolutionary relationships of the Punung sample. Either some of the specimens could belong to the same taxa as the fossils from Vietnam and China (assigned to *P*. *devosi* and *P*. *weidenreichi*, respectively), or they could represent an endemic taxon (cf. *P*. *javensis*) as proposed by Drawhorn [11]. The former assumption would lead to a scenario where a group close to *P*. *weideinreichi*/*P*. *devosi* evolved in Java leading to a dental reduction compared to the continental populations but remaining morphologically and genetically compatible with them. In the second scenario, they would have evolved into a separate geographical variant. Based on the morphospecies concept, as commonly used in paleontological studies, the distinct range of EDJ morphology of a great part of this Punung sample (vs China and Vietnam) would indicate the existence of this endemic taxon (*P*. *javensis*).

Size reduction could be either the result of an evolutionary trend in insular environments due to limited resources, or the result of the founder effect of a population of small-sized individuals. The Punung fossil-bearing layers were deposited between 128 ka and 117 ka which corresponds to the early period of the Last Interglacial, when Java might still have had some remaining connections to neighboring land masses, which favored the expansion range of *Pongo* [57, 58]. This timing is consistent with paleoclimatic records for the western region of Java, which indicate shifts towards very warm and humid conditions c. 126 ka in the Bandung basin, Java [58, 87, 88]. However, other reconstructions of global sea level fluctuations also suggest sea level rises around 120 ka separating Java from continental Asia, Borneo and Sumatra [89], and it was proposed that these insular conditions might have caused a selective pressure in favor of dental size reduction [5, 85]. A more extensive comparative modern and fossil dataset is required to verify, if fossils from Punung, showing an affinity to continental Southeast Asian fossils, are a distinct paleospecies or fall within the modern species range of variation.

Further samples from Indonesia such as the Early to Middle Pleistocene *Pongo* remains from the Sangiran Dome are the oldest known fossils from the Sunda region (dated to 1.5–0.5 Ma). It is likely that the orangutan populations like that recorded at Sangiran were widespread across Sundaland in the Middle Pleistocene. These populations are considerably older than the Punung fossils. The typicality probabilities groups for SMF 8858 and SMF 8864 show affinities to all fossil groups with the latter demonstrating a high affinity to Punung fossils. SMF 8879 has an affinity to *P*. *abelii* and needs to be investigated more closely. The typicality probabilities of the Sangiran samples are not uniform, which suggests a high variability.

However, the small sample size complicates the assignment and characterization of *Pongo* from the Sangiran Dome and its relationship to other fossils from continental Southeast Asia and Sunda. It is unclear if *Pongo* from the subsequent Punung site could be either a continuation of *Pongo* from Sangiran, both located on Java, or the result of a later expansion from Late Pleistocene continental Southeast Asia. Our data do not provide a conclusive statement regarding the relationship of *Pongo* from Punung and Sangiran. A re-examination focusing on the Punung fossil collections is required, in order to separate the possibly terminal Pleistocene to Holocene material from the early Late Pleistocene specimens, which might convolute the morphometric and phylogenetic characterization of the Punung material.

The specimen S2258 from the Late Pleistocene Niah Cave, Borneo shows a high affinity to fossils from Vietnam based on typicality probabilities (Table 3). This tooth was previously reported as identical in size and morphology to modern *P*. *pygmaeus* [6]. More specimens are needed to test the relationship between Niah Cave samples, Vietnamese samples and *Pongo pygmaeus*.

#### 4.1.4 Fossil *Pongo* from mainland Asia

Fossils from China, Baikong and Mohui cave dated to 2.2–2.0 Ma are the oldest known fossils identified as *Pongo* [6, 85, 90, 91]. Hooijer [1] assigned Early to Middle Pleistocene orangutans discovered in southern China to *P*. *pygmaeus weidenreichi* based on the large size of teeth. Harrison et al. [6] elevated *Pongo weidenreichi* to species level based on morphometric traits such as lower crowned canines and relatively large second and third molars compared to modern species. Our results show no overlap between the range of *P*. *pygmaeus* and that of fossil molars from China but little overlap with *P*. *abelii*. Futhermore, most of the fossils from China cluster on the negative morphospace along CV1 with a higher affinity to fossils from Vietnam.

Fossil teeth from Chinese sites bought in drug stores at Manila and Hong Kong by von Koenigswald [39, 40] were allocated to several taxa such as *Hemanthropus peii*, *Sinanthropus officinalis* (*Homo erectus*) and fossil *Pongo*. The *Pongo* teeth identified are thought to have come from Pleistocene caves in southern China based on preservation and associated faunas. These teeth are described as larger than those of modern *Pongo* [1, 39]. However, specimens CA805, CA806 and CA807 were originally assigned to *Sinanthropus officinalis* [40]. Smith et al. [26] reevaluated the taxonomy of isolated teeth from the Chinese apothecary collection and showed a correspondence of these specimens to *Pongo* based on dental periodicity, EDJ shape and enamel thickness. Only CA806 was assigned to “likely *Homo*” by Smith et al. [26] due to the low periodicity of 8 days, resembling periodicities reported in *Homo* while *Pongo* shows higher periodicities. But it was noted by Smith et al. [26] that the EDJ shape resembles *Pongo* rather than *Homo*. Our results confirm that specimens from the Chinese apothecary collection share the same morphospace as specimens from Ganxian (identified as *P*. *weidenreichi*; [38]). Although, Chinese samples are showing a high variation along CV3 with several specimens from Ganxian grouping closer to Vietnamese fossils, the others group closer with molars from the Chinese Apothecary Collection. This is reflected in the cross-validated classification results for Chinese specimens as well, which demonstrates a high affinity to fossils from Vietnam. Nevertheless, if CV1/CV2 is considered, Ganxian and Chinese apothecary collection fossils group close together. Hence, all fossils from China could belong to a similar morphotype, even if *Pongo* from the Chinese Apothecary collection is difficult to characterize due to the lack of the exact provenance and age.

Schwartz et al. [3] examined fossils from various Middle to Late Pleistocene sites in Vietnam and established several (sub)species such as *P*. *hooijeri* (Tham Khuyen), *P*. *pygmaeus ciochoni* (Lang Trang), *P*. *pygmaeus devosi* (Hang Hum), *P*. *pygmaeus kahlkei* (Tham Khuyen) and *P*. *pygmaeus fromageti* (Tham Om). They also described a new ape genus *Langsonia* in northern Vietnam. Cameron [92] argued that the large mainland taxa (from China and Vietnam) cluster together and are separated from smaller island taxa of Borneo and Sumatra. Following Schwartz et al. [3], Cameron [92] confirmed the distinctiveness of *P*. *p*. *ciochoni* (Lang Trang) from other *Pongo* taxa. Our results, however, show morphological similarities of all fossils from Vietnam as they cluster together in one morphospace including *P*. *p*. *ciochoni*.

It was suggested that the diversity observed by Schwartz [25] reflects the high morphological variability within Pleistocene *Pongo* rather than multiple (sub)species or a new genus [4, 65]. Later, Harrison et al. [6] reexamined the (sub)species created by Schwartz et al. [3] and concluded that there are not sufficient morphological differences to justify these (sub)species or a new genus. This interpretation is supported by our result, although the identification of subspecies in the fossil record might be difficult, considering that a distinction at the species level is already confused by various conflicting species distinctions [1–4, 6, 7, 11, 56, 83].

Harrison et al. [6] allocated Middle Pleistocene specimens from China and Vietnam to *P*. *weidenreichi* and Late Pleistocene specimens from the same area to *P*. *devosi*, thus re-allocating all (sub)species defined by Schwartz et al. [3] to junior synonyms. *P*. *devosi* is described as relatively smaller than *P*. *weidenreichi* and might have replaced the latter during the Late Pleistocene. It was recently debated whether *P*. *devosi* should be considered as a distinct species or a junior synonym to *P*. *weidenreichi* based on a bigger sample size of fossil orangutan teeth from China [7]. In their study, Harrisonet al. [7] proposed that *P*. *weidenreichi* and *P*. *devosi* may be regarded as one single species since *P*. *weidenreichi* exhibited a temporal trend of size decrease in the Late Pleistocene. Furthermore, it was argued that throughout the Early to Late Pleistocene, *Pongo*’s distinctive morphological features remained unaltered, justifying the demotion of *P*. *devosi* to a synonym of *P*. *weidenreichi* [7, 93]. Our results indicate substantial overlap between fossils from China and Vietnam along CV1 vs. CV2 (Fig 1A), also indicated by the cross-validated classification results (S2 Table), but with some morphometric distinction along CV3. More Chinese specimens are needed to determine whether *P*. *devosi* and *P*. *weidenreichi* represent a single chronospecies or separate species.

The greater size of Early to Middle Pleistocene *Pongo* fossils from China compared to their modern counterparts and the temporal trend of diminution of tooth size from the Pleistocene to the Holocene were observed in several studies [83, 92, 94, 95]. Other authors show that reduction in tooth size throughout the Quaternary might not strictly follow the chronology [5, 51, 85, 92]. This is in line with our results indicating that shape changes in the EDJ are not mainly tied to dental size.

Fossil *Pongo* specimens from Tham Prakai Phet (Thailand) dated to the Late Pleistocene show a bigger size compared to *P*. *devosi*, suggesting an affinity to the species *P*. *weidenreichi* [51]. We observe the same trend for other fossils from the mainland. Specimens from the Late Pleistocene Batu Cave (66–33 ka) in Peninsular Malaysia are larger than specimens from the Middle Pleistocene site of Badak Cave C (500 ka) proving that *Pongo* did not experience a steady decline in tooth size over time. As previously hypothesized [51], two forms of *Pongo* might have existed during the Late Pleistocene, a big morph in the center of the continent (represented by the Thai and Malay specimens) and a smaller one represented by Vietnamese specimens assigned to *P*. *devosi*.

*Pongo* remains from the Late Pleistocene site in Laos (Tam Hay Marklot Cave) were reported as *Pongo* sp. [45, 48, 50]. Our results show that the specimen “ML5” shows a high affinity to the *P*. *abelii* sample and to fossils from the same area in Vietnam (Table 3). Due to the limited sample size, a definite assignment is not feasible. Additional samples are required to clarify the relationships between, on one hand, Late Pleistocene *Pongo* from Tam Hay Marklot and *P*. *abelii*, and on the other hand, the relationships between fossils from the mainland.

In conclusion, there is morphometric variation within fossil *Pongo* from continental SEA, pointing to the distinction of fossil morphotypes of *Pongo* compatible with the previously published species *P*. *weidenreichi* and *P*. *devosi*. Whether the Punung specimens represent a mix of early Late Pleistocene attributable to *P*. *weideinreichi*/*P*. *devosi* or *P*. *javensis* and later specimens (terminal Pleistocene to Holocene) related to the living species of *Pongo* remains unclear. The Punung assemblage is the only one in our fossil sample, which is represented in a large number of specimens from one specific locality. This might be the reason for the higher classification results as opposed to fossils from Vietnam and China.

### 4.2 Proteomic challenges in *Pongo* species distinction

The preserved protein regions were not informative to confidently discriminate between the different species of *Pongo*. There are no species-specific taxonomic markers that may be derived from our experimental protocols. Inspection of multiple sequence alignments, including a broad selection of *in silico* sequences from hominids such as *Pan* and *Gorilla*, reveals that these enamel protein sequences display a smaller intrageneric variability in *Pongo* than in other hominids. For instance, chimpanzees (*Pan*) display several taxonomic markers within frequently preserved regions, enabling the differentiation of extant species. This accounts for the clear separation between *Pan troglodytes* and *Pan paniscus* in our tree (Fig 5). It is not clear what is driving this lack of variation in enamel protein sequences among extant *Pongo* species. Recent DNA analyses revealed several admixture events resulting in gene flow between populations throughout the Pleistocene, which ceased completely 20–10 ka due to habitat loss in areas between the species’ ranges and isolation on islands [17]. The lack of variation in protein regions might be a consequence of high admixture within the genus *Pongo*. Biogeographical events played a key role in facilitating exchange between *Pongo* populations from the mainland Southeast Asia and the Sunda region (see below 4.3.) [96, 97].

All extant *Pongo* species share a single SAP at position 174 in AMBN, which is not present in two fossil *Pongo* specimens (one from the Sangiran site and one from the Duoi U’Oi site). Fossil *Pongo* displays the same SAP version found in all available great ape taxa, setting them apart from modern *Pongo*. However, interpreting whether this SAP demonstrates that extant *Pongo* might be evolutionarily derived compared to fossil *Pongo* from the Early to Middle Pleistocene should be approached with caution. This is due to its limited occurrence in our fossil dataset and its constrained significance for broader evolutionary conclusions, as evidenced by the grouping of all *Pongo* fossils and modern specimens in the same clade (Fig 5).

Our protein translations from the genomic data of modern *Pongo* individuals show that even the full coverage of the enamel proteins cannot clearly separate between the different extant species. This is intriguing, considering that distinguishing between extant *Pan* and *Gorilla* species at the enamel protein level is possible. The enamel proteins were also unable to identify groupings within the fossil *Pongo* specimens. Separation between extant and fossil *Pongo* might be possible with a higher coverage however, given the identification of at least one separating SAP, or through the recovery of a richer proteome, for example from bone or dentine. Similar to *Pongo*, within the genus *Homo*, enamel proteins reveal less information for determining closely related hominin species [33]. This discrepancy in phylogenetic resolution within dental enamel proteins among African great apes, *Pongo*, and *Homo* need further investigation.

The EDJ analysis can identify different groups within both the extant and the fossil *Pongo* sample. This implies that EDJ morphometrics are a more viable method for distinguishing groups of closely related primates, where the enamel proteome offers limited resolution. However, in cases where dental fossil specimens exhibit incomplete morphology due to damage from fossilization processes, the analysis of dental enamel from fossil *Pongo* specimens could provide opportunities to identify the genus at least. This is particularly important in fossil sites like Sangiran, where a high diversity of hominid species was confirmed including fossil *Pongo*, *Homo erectus*, *Meganthropus* and possibly *Gigantopithecus* [27]. The convergence in molar outer crown morphology adds complexity to taxonomic classifications, especially when dealing with fragmented specimens. The protein sequences can be utilized in conjunction with the EDJ analysis to confidently place those samples within a wider phylogenetic framework.

### 4.3 Paleoenvironmental implications on the speciation of *Pongo*

Sundaland emerged as a continuous landmass connecting the Indonesian archipelago with continental Southeast Asia during the Gelasian Stage (2.59–1.81 Ma). Land bridge formations between Sundaland and mainland Asia during the Early Pleistocene facilitated colonization of these regions by mammalian taxa [88, 98–100]. Paleogeographic changes and paleoclimatic oscillations related to spreading and declining of rainforest habitats likely impacted the evolution and speciation of *Pongo* in Southeast Asia [97, 101]. During the Pleistocene, cool and dry periods which led to a decline and fragmentation in rainforest habitats and the emergence of open savannahs, alternated with warm and wet periods with sea level rises temporarily disconnecting islands and isolating taxa [96–98, 102–106]. Repeated fluctuations of sea levels contributed to either isolation phases of islands from the Sunda Shelf during which isolated populations of orangutans evolved differently from the continental and other island groups, or intermittent exchanges between continental and insular populations during low sea level events [97]. Sundaland was marked by its low relief and several large rivers. Fragmentation of rainforests due to increase in drier conditions and large rivers dissecting Sundaland hindered the dispersal of orangutan populations and potentially decreased population sizes [97, 101]. It was hypothesized that the formation of a large savannah corridor through Sundaland, starting from the eastern Sumatran highlands and continuing to western Borneo [98], might have been an additional barrier for orangutan dispersal. The emergence of a savannah corridor probably caused the formation of forest refugia in the highlands of Java and Sumatra and northeastern and southern Borneo [98]. Due to these conditions populations of orangutans probably remained ecologically/geographically separated, increasing the probability of genetic drift and local variations. These conditions might have contributed to the high genetic and phenotypic variation of *Pongo* during the Pleistocene. The divergence of *P*. *tapanuliensis* and *P*. *abelii* both from Sumatra are reported as 3.38 Ma, which is longer than the divergence of *P*. *pygmaeus* from Borneo and *P*. *abelii* [17]. Nevertheless, gene flow occurred occasionally after the separation of these species during the Pleistocene affecting the genetic and phenotypic/morphological variation of *Pongo*. Indeed genetic evidence revealed repeated gene flow events between *P*. *abelii*, *P*. *pygmaeus* and *P*. *tapanuliensis* throughout the Pleistocene [17]. This demonstrates the complex evolutionary history of *Pongo* and the need for DNA analyses on fossil material to disentangle the evolutionary relationships between fossils from Indochina and insular Southeast Asia and to determine their relationships to modern species.

## 5. Conclusions

EDJ morphology shows tentative support for previously established fossil *Pongo* species, which were solely based on linear measurements and morphometric traits of teeth. Our separate analyses for M1/M2s and M3s, along with the combined analysis, highlight that despite the issue of molar position and sample size, (1) fossil *Pongo* from Vietnam, Indonesia and China show differences to modern species and (2) fossils from Indonesia are different in their EDJ shape to fossils from Vietnam and China. It is clear that our morphometric results are constrained by the small sample size by site and by period (ML5, Tam Hay Marklot, Laos; SMF 8879, SMF 8864, SMF 8858, Sangiran Dome, Java; S2258, Niah Cave, Malaysian Borneo), which is not sufficient to estimate the variability of past populations and their relationships.

The Punung specimens might represent a mix of early Late Pleistocene and later specimens (terminal Pleistocene to Holocene) closely related to extant *Pongo*. These Late Pleistocene specimens show morphometric differences to fossils from continental Southeast Asia, whether these differences might be attributable to a separate species (such as *P*. *javensis*) has to be confirmed with further analyses.

Fossils from Vietnam and China show some morphometric separation, but substantial overlap exist between groups. Larger sample sizes are required to adequately test whether *P*. *weidenreichi* (China) and *P*. *devosi* (Vietnam) species represent two distinct species or belong to one chronospecies. Paleoproteomic data from dental enamel proteins do not permit a consistent attribution of fossil specimens to either *P*. *pygmaeus* or *P*. *abelii*, but avoid debates about the appurtenance of isolated fossil specimens to *Pongo* (versus *Homo*) with a direct (secure, robust) assignment to the genus [3, 43]. In that respect, EDJ morphometry shows some separation in closely related fossil groups within a genus, where the enamel proteome offers limited resolution. Nevertheless, protein sequences can be utilized with EDJ morphology to confidently place these fossil samples in a broader phylogenetic framework, including other hominid taxa.

Orangutans can be a model for the understanding of human evolution due to known hybridization between modern *Pongo* species, the high morphological variability in dental remains and the large fossil record, which is unique among other great apes such as *Gorilla* and *Pan*. Analyses of the evolutionary history of *Pongo* and their paleoenvironments are crucial to improve conservational efforts for critically endangered species of *Pongo*.

## Supporting information

S1 Table List of specimens used in the present study. (PDF)

S2 Table Cross-validated classification results in frequencies based on M1–M2. (PDF)

S3 Table Cross-validated classification results in percentages based on M1–M2. (PDF)

S4 Table Cross-validated classification results in frequencies based on M3. (PDF)

S5 Table Cross-validated classification results in percentages based on M3. (PDF)

S6 Table Summary statistics of paleoproteomic analyses. (PDF)

S7 Table Semilandmark coordinates for each specimen used in this study. (CSV)

S1 Fig **a** Cross-validated CVA of the EDJbased on M1–M2. Jackknife cross-validation was performed using the same groups as in the CVA (Fig 1A) to validate group discrimination in Fig 1A. **b** Cross-validated CVA of the EDJ based on M3s. Jackknife cross-validation was performed using the same groups as in the CVA (Fig 1B) to validate group discrimination in Fig 1B.
(ZIP)

S2 Fig Maximum likelihood phylogenetic tree. Maximum Likelihood phylogenetic tree created by using PhyML based on protein sequences of several great ape species (*Gorilla gorilla*, *Gorilla beringei graueri*, *Pan paniscus*, *Pan troglodytes schweinfurthii*, *Pan troglodytes ellioti*, *Pongo pygmaeus*, *Pongo abelii*, *Pongo tapanuliensis* and *Homo sapiens*) and fossil *Pongo* samples included. *Nomascus leucogenys* was added as an outgroup. In this tree both modern and fossil *Pongo* are placed within the same clade but with moderate support. Some substructure can be observed on the tree but without any concrete support.
(TIF)

S3 Fig Molar type assignments. Heatmap of molar type assignments by three observers (JK, CZ, AMB) on a subsample of isolated *Pongo* fossil molars. Dark blue indicates consent by all 3 observers, medium blue indicates consent among 2 observers, light blue shows one observer assigned the molar to the specific position and light grey means no assignment.
(TIF)

S4 Fig CVA of the EDJ based on all molar positions combined. (TIF)

S5 Fig Cross-validated CVA of the EDJ based on all molar positions combined. (TIF)

S6 Fig a PCA analyses of the EDJbased on M1–M2. b PCA analyses of the EDJ based on M3. c PCA analyses of the EDJ based on all molar positions.
(ZIP)

S1 File Palaeoproteomic phylogenetic analysis. (DOCX)

S1 Data (CSV)
