# Supplementary material for: Geometric morphometrics and paleoproteomics enlighten the paleodiversity of Pongo
Source: PLoS One. 2023 Dec 15;18(12):e0291308. doi: 10.1371/journal.pone.0291308 (PMC10723683; doi:10.1371/journal.pone.0291308)
Supplement: S1 Table — (PDF) [file pone.0291308.s001.pdf]

S1 Table. List of specimens used in the present study.

Proven-ance	Site	Specimen ID	n	Tooth type	Catalogue ID	Geometric morphometrics	Paleo-proteomics	Curator
China	Ganxian	GX6002, GXB6005, GXI007, GX010, GX3012	5	Lower molar	GX-6002, GX-B6005, GX-I007, GX-010, GX-3012	GX6002, GXB6005, GXI007, GX010, GX3012		wangw@sdu.edu.cn
China	Chinese Apothecary Collection	CA260, CA805, CA806, CA807	4	Lower molar	CA 260, CA 805, CA806, CA 807	CA260, CA805, CA806, CA807		ottmar.kullmer@senckenberg.de
Vietnam	Tham Om	75TO17, 75TO22, 77TO9bv49, 77TOo1335, 77TOo1428, 77TOo7bv38, 77TOo930, 77TOv0139	8	Lower molar	75.TO.17, 75.TO.22, 77.TO.9b.v.49, 77.TO.o.13.35, 77.TO.o.14.28, 77.TO.o.7b.v.38, 77.TO.o.9.30, 77.TO.v.01.39	75TO17, 75TO22, 77TO9bv49, 77TOo1335, 77TOo1428, 77TOo7bv38, 77TOo930, 77TOv0139		maihuong72@gmail.com; nguyenanhtuan_bio@yahoo.com.vn
Vietnam	Hoà Binh	S1181	1	Deciduous canine	S1181:Noi Sang		S1181	maihuong72@gmail.com; nguyenanhtuan_bio@yahoo.com.vn
Vietnam	Lang Trang	93LT111M1l, 93LT88, 93LT89, 93LT91, 93LTC2B5108M1l, 93LTC2B5126M12l, 93LTC2B5131, 93LTC2B5132, 93LTC2B597, 93LTC2B598	10	Lower molar	93.LT.111, 93.LT.88, 93.LT.89, 93.LT.91, 93.LT.C2B5.108, 93.LT.C2B5.126, 93.LT.C2B5.131, 93.LT.C2B5.132, 93.LT.C2B5.97, 93.LT.C2B5.98	93LT111M1l, 93LT88, 93LT89, 93LT91, 93LTC2B5108M1l, 93LTC2B5126M12l, 93LTC2B5131, 93LTC2B5132, 93LTC2B597, 93LTC2B598		maihuong72@gmail.com; nguyenanhtuan_bio@yahoo.com.vn
Vietnam	Duoi U'Oi	DU996, DU998, DU1177, DU1012, DU1128,	4	Lower molar	DU996, DU998, DU1177, DU1012, DU1128	DU996, DU998, DU1177, DU1012	DU1128, DU1177	maihuong72@gmail.com; nguyenanhtuan_bio@yahoo.com.vn
Vietnam	Hung Hum	HH42M2lR, HH55M2lR	2	Lower molar	HH42, HH55	HH42M2lR, HH55M2lR		maihuong72@gmail.com; nguyenanhtuan_bio@yahoo.com.vn
Vietnam	Mai Da Dieu	MDD10-M3lL	`	Lower molar	MDD10	MDD10-M3lL		maihuong72@gmail.com; nguyenanhtuan_bio@yahoo.com.vn
Vietnam	Thung Lang	TL001, TL002, TL4, TL17, TL18, TL30, TL36, TL37, TL38, TL39	10	Lower molar	TL001, TL002, TL4, TL17, TL18, TL30, TL36, TL37, TL38, TL39	TL001, TL002, TL4, TL17, TL18, TL30, TL36, TL37, TL38, TL39		maihuong72@gmail.com; nguyenanhtuan_bio@yahoo.com.vn

Laos	Marklot	ML5, S1113, ML1, ML4, ML6	1	ML5 = Lower left M1 S1113 = upper right P4 ML1= lower right M1 ML4= upper left P4 ML6= upper right M1	ML5, S1113, ML1, ML4, ML6	ML5	ML5, S1113, ML1, ML4, ML6	viengkaev@gmail.com
Laos	Tam Hang	TH929	1	Upper molar			TH929	viengkaev@gmail.com
Thailand	Tham Prakai Phet	TPKP-NE-128	1	Lower left P4	TPKP-NE-128		TPKP-NE-128	essathiwat@gmail.com
Malaysia	Niah Cave	S2258, S2246, S2249, S2254	1	S2258 = lower molar, S2249 =Upper jaw fragment including dentition	S2258: EW E 45-48 S2246: Y/2A S2249: Y/3 48-54 S2254 : 36-48	S2258	S2258, S2246, S2249, S2254	limtzetshen@yahoo.com
Indonesia	Punung	PU-1, PU-3, PU-5, PU-8, PU-4, PU-12, PU-13, PU-14, PU-15, PU-16, PU-20, PU-22, PU-27, PU-29, PU-35, PU-39, PU-44, PU-181, PU-182, PU-186, PU-191, PU-195	22	Lower molar	PU 1, PU 3, PU 5, PU 8, PU 4, PU 12, PU 13, PU 14, PU 15, PU 16, PU 20, PU 22, PU 27, PU 29, PU 35, PU 39, PU 44, PU 181, PU 182, PU 186, PU 191, PU 195	PU-1, PU-3, PU-5, PU-8, PU-4, PU-12, PU-13, PU-14, PU-15, PU-16, PU-20, PU-22, PU-27, PU-29, PU-35, PU-39, PU-44, PU-181, PU-182, PU-186, PU-191, PU-195		ottmar.kullmer@senckenberg.de
Indonesia	Sangiran	SMF-8858, SMF-8864, SMF-8879	3	Lower molar	SMF-PA-F-8858, SMF-PA-F-8864, SMF-PA-F-8879	SMF-8858, SMF-8864, SMF-8879	SMF-8864	ottmar.kullmer@senckenberg.de
Pongo abelii		Icet163LLM1, MAM7360M1, MAM7360M2, MAM7360M3, MAM7361M1, MAM7361M2, MAM7361M3, SMF59148LLM2 SMF59148LLM3 SMF59148LRM2 SMF59148LRM3 ZMB1209M1, ZMB1209M2, ZMB1209M3, ZMB67173M1, ZMB67173M2, ZMB67173M3,	20	Lower M1, M2 and M3	Icet163, MAM7360, MAM7361, SMF59148, ZMB1209, ZMB67173, ZMB83509	Icet163LLM1, MAM7360M1, MAM7360M2, MAM7360M3, MAM7361M1, MAM7361M2, MAM7361M3, SMF59148LLM2 SMF59148LLM3 SMF59148LRM2 SMF59148LRM3 ZMB1209M1, ZMB1209M2, ZMB1209M3, ZMB67173M1, ZMB67173M2, ZMB67173M3,		Icet163 (https://www.morphosource.org/) MAM7360, MAM7361, ZMB1209, ZMB67173, ZMB83509 jean-jacques.hublin@college-de-france.fr SMF59148,

		ZMB83509M1, ZMB83509M2 ZMB83509M3				ZMB83509M1, ZMB83509M2 ZMB83509M3		ottmar.kullmer@ senckenberg.de
Pongo pygmaeus		SMF16745LLM3 SMF16745LRM1 SMF16745LRM2 SMF16745LRM3 SMF2639LLM1, SMF2639LLM2, SMF2639LRM1, SMF2639LRM2, ZMB30944M1, ZMB30944M2, ZMB30944M3, ZMB38607M1, ZMB38607M2, ZMB38607M3, ZMB6948M1, ZMB6948M2, ZMB6948M3, ZMB6954M1, ZMB6954M2, ZMB6954M3, ZMB7875M1, ZMB7875M2, ZMB7875M3	23	Lower M1, M2 and M3	SMF16745, SMF2639, ZMB30944, ZMB38607, ZMB6948, ZMB6954, ZMB7875	SMF16745LLM3 SMF16745LRM1 SMF16745LRM2 SMF16745LRM3 SMF2639LLM1, SMF2639LLM2, SMF2639LRM1, SMF2639LRM2, ZMB30944M1, ZMB30944M2, ZMB30944M3, ZMB38607M1, ZMB38607M2, ZMB38607M3, ZMB6948M1, ZMB6948M2, ZMB6948M3, ZMB6954M1, ZMB6954M2, ZMB6954M3, ZMB7875M1, ZMB7875M2, ZMB7875M3		ZMB30944, ZMB38607, ZMB6948, ZMB6954, ZMB7875 jean-jacques.hublin@college-de-france.fr<br><br>SMF16745, SMF2639, ottmar.kullmer@senckenberg.de
