# Supplementary material for: Geometric morphometrics and paleoproteomics enlighten the paleodiversity of Pongo
Source: PLoS One. 2023 Dec 15;18(12):e0291308. doi: 10.1371/journal.pone.0291308 (PMC10723683; doi:10.1371/journal.pone.0291308)
Supplement: S2 Table — (PDF) [file pone.0291308.s002.pdf]

S2 Table. Cross-validated classification results in frequencies.

	China	Indonesia	*P. abelii*	*P. pygmaeus*	Vietnam
China	4/9	1/9	1/9	0/9	3/9
Indonesia (Punung)	3/22	14/22	0/22	2/22	0/22
P. abelii	1/13	0/13	10/13	1/13	1/13
P. pygmaeus	0/16	2/16	3/16	11/16	0/16
Vietnam	2/24	5/24	1/24	1/24	15/24
