# Supplementary material for: Geometric morphometrics and paleoproteomics enlighten the paleodiversity of Pongo
Source: PLoS One. 2023 Dec 15;18(12):e0291308. doi: 10.1371/journal.pone.0291308 (PMC10723683; doi:10.1371/journal.pone.0291308)
Supplement: S3 Table — (PDF) [file pone.0291308.s003.pdf]

S3 Table. Cross-validated classification results in percentages.

	China	Indonesia	*P. abelii*	*P. pygmaeus*	Vietnam
China	44.44%	11.11%	11.11%	0.00%	33.33%
Indonesia	15.79%	73.68%	0.00%	10.53%	0.00%
P. abelii	7.69%	0.00%	76.92%	7.69%	7.69%
P. pygmaeus	0.00%	12.5%	18.75%	68.75%	0.00%
Vietnam	8.33%	20.83%	4.17%	4.17%	62.50%
overall classification accuracy:	66.67%				
