# Supplementary material for: Geometric morphometrics and paleoproteomics enlighten the paleodiversity of Pongo
Source: PLoS One. 2023 Dec 15;18(12):e0291308. doi: 10.1371/journal.pone.0291308 (PMC10723683; doi:10.1371/journal.pone.0291308)
Supplement: S4 Table — (PDF) [file pone.0291308.s004.pdf]

S4 Table. Cross-validated classification results in frequencies for M3

	Indonesia	*P. abelii*	*P. pygmaeus*	Vietnam
Indonesia (Punung)	5/6	0/6	0/6	1/6
P. abelii	0/7	3/7	2/7	2/7
P. pygmaeus	2/7	1/7	3/7	1/7
Vietnam	2/12	3/12	2/12	5/12
