# Supplementary material for: Geometric morphometrics and paleoproteomics enlighten the paleodiversity of Pongo
Source: PLoS One. 2023 Dec 15;18(12):e0291308. doi: 10.1371/journal.pone.0291308 (PMC10723683; doi:10.1371/journal.pone.0291308)
Supplement: S5 Table — (PDF) [file pone.0291308.s005.pdf]

S5 Table. Cross-validated classification results in percentages for M3.

	Indonesia	*P. abelii*	*P. pygmaeus*	Vietnam
Indonesia	83.33%	0.00%	0.00%	16.67%
P. abelii	0.00%	42.86%	28.57%	28.57%
P. pygmaeus	28.57%	14.29%	42.86%	14.29%
Vietnam	16.67%	25.00%	16.67%	41.67%
overall classification accuracy:	50%			
