# Supplementary material for: Geometric morphometrics and paleoproteomics enlighten the paleodiversity of Pongo
Source: PLoS One. 2023 Dec 15;18(12):e0291308. doi: 10.1371/journal.pone.0291308 (PMC10723683; doi:10.1371/journal.pone.0291308)
Supplement: S6 Table — (PDF) [file pone.0291308.s006.pdf]

S6 Table. Summary statistics of palaeoproteomic analyses.

Protein	Sample	Source	Razor+ Unique peptides	# of amino acids covered	Coverage (%)	Deamidation N (% ± 1 SD)	Deamidation Q (% ± 1 SD)
KRT9	Lab Blank #1	Contaminant	1	9	1.44	100.0 (0.0)	100.0 (0.0)
AMBN	Lab Blank #1	Enamel proteome	5	14	3.13	N/A	100.0 (0.0)
KRT1	Lab Blank #2	Contaminant	6	49	7.60	100.0 (0.0)	100.0 (0.0)
KRT9	Lab Blank #2	Contaminant	2	9	1.44	100.0 (0.0)	100.0 (0.0)
ENAM	Lab Blank #2	Enamel proteome	1	12	1.05	N/A	N/A
ALB	Lab Blank #2	Enamel proteome	3	11	1.81	N/A	N/A
KRT9	Lab Blank #3	Contaminant	10	84	13.46	N/A	N/A
KRT1	Lab Blank #3	Contaminant	26	171	26.51	N/A	N/A
KRT2	Lab Blank #3	Contaminant	1	8	1.23	N/A	N/A
KRT17	Lab Blank #3	Contaminant	4	36	8.31	N/A	N/A
ALB	Lab Blank #3	Enamel proteome	2	23	3.79	N/A	N/A
ALB	Lab Blank #4	Enamel proteome	3	17	2.80	N/A	N/A
COL3A1	TPKP-NE-128	Contaminant	1	13	0.89	100.0 (0.0)	N/A
AHSG	TPKP-NE-128	Contaminant	1	14	3.88	0.0 (0.0)	N/A
KRT78	TPKP-NE-128	Contaminant	2	15	2.87	N/A	N/A
AFM	TPKP-NE-128	Contaminant	1	12	1.98	100.0 (0.0)	100.0 (0.0)
AMEL	TPKP-NE-128	Enamel proteome	472	168	81.95	97.4 (1.7)	98.1 (0.7)
AMBN	TPKP-NE-128	Enamel proteome	207	155	34.68	99.9 (0.0)	98.6 (0.8)

ENAM	TPKP-NE-128	Enamel proteome	367	188	16.46	97.3 (1.0)	95.6 (1.4)
MMP20	TPKP-NE-128	Enamel proteome	2	18	3.73	100.0 (0.0)	N/A
AMTN	TPKP-NE-128	Enamel proteome	0	0	0	N/A	100.0 (0.0)
ALB	TPKP-NE-128	Enamel proteome	3	8	1.31	100.0 (0.0)	N/A
COL17A1	TPKP-NE-128	Enamel proteome	9	38	2.54	N/A	N/A
AHSG	ML1	Contaminant	1	14	3.88	0.0 (0.0)	N/A
KRT2	ML1	Contaminant	1	8	1.23	100.0 (0.0)	N/A
KRT78	ML1	Contaminant	1	7	1.34	100.0 (0.0)	N/A
AFM	ML1	Contaminant	2	12	1.98	100.0 (0.0)	100.0 (0.0)
AMEL	ML1	Enamel proteome	502	180	87.80	98.1 (0.9)	97.5 (0.7)
AMBN	ML1	Enamel proteome	289	195	43.62	99.9 (0.0)	98.7 (0.6)
ENAM	ML1	Enamel proteome	446	212	18.56	97.6 (0.7)	96.6 (1.0)
MMP20	ML1	Enamel proteome	7	43	8.90	100.0 (0)	N/A
AMTN	ML1	Enamel proteome	39	43	20.57	N/A	97.7 (2.0)
ALB	ML1	Enamel proteome	19	76	12.48	97.9 (2.1)	100.0 (0)
COL17A1	ML1	Enamel proteome	42	96	6.41	N/A	100.0 (0)
COL3A1	TH929	Contaminant	1	13	0.89	100.0 (0.0)	N/A
AHSG	TH929	Contaminant	1	14	3.88	0.0 (0.0)	N/A
KRT2	TH929	Contaminant	1	8	1.23	0.0 (0.0)	N/A
KRT78	TH929	Contaminant	2	15	2.87	100.0 (0.0)	N/A
AFM	TH929	Contaminant	2	12	1.98	100.0 (0.0)	100.0 (0.0)
AMEL	TH929	Enamel proteome	504	168	81.95	97.6 (1.3)	98.5 (0.5)
AMBN	TH929	Enamel proteome	245	173	38.70	99.9 (0.0)	98.3 (0.8)
ENAM	TH929	Enamel proteome	367	211	18.48	96.0 (1.5)	95.6 (1.3)
MMP20	TH929	Enamel proteome	6	39	8.07	100.0 (0.0)	100.0 (0.0)
AMTN	TH929	Enamel proteome	2	15	7.18	N/A	100.0 (0.0)
ALB	TH929	Enamel proteome	4	16	2.63	100.0 (0.0)	N/A
COL17A1	TH929	Enamel proteome	19	61	4.07	N/A	100.0 (0.0)
COL3A1	ML4	Contaminant	1	13	0.89	100.0 (0.0)	N/A
KRT17	ML4	Contaminant	1	12	2.77	N/A	0.0 (0.0)
AHSG	ML4	Contaminant	1	14	3.88	0.0 (0.0)	N/A
KRT2	ML4	Contaminant	1	8	1.23	100.0 (0.0)	N/A
KRT78	ML4	Contaminant	2	15	2.87	100.0 (0.0)	N/A
AFM	ML4	Contaminant	2	21	3.47	100.0 (0.0)	48.9 (37.6)

AMEL	ML4	Enamel proteome	571	183	89.27	96.1 (1.5)	97.2 (0.8)
AMBN	ML4	Enamel proteome	347	214	47.87	99.8 (0.1)	98.3 (0.8)
ENAM	ML4	Enamel proteome	491	229	20.05	97.8 (0.7)	96.6 (0.9)
MMP20	ML4	Enamel proteome	4	45	9.32	100.0 (0.0)	100.0 (0.0)
AMTN	ML4	Enamel proteome	2	13	6.22	N/A	100.0 (0.0)
ALB	ML4	Enamel proteome	2	15	2.46	100.0 (0.0)	N/A
COL17A1	ML4	Enamel proteome	38	118	7.88	N/A	100.0 (0.0)
COL3A1	ML5	Contaminant	1	13	0.89	100.0 (0.0)	N/A
KRT1	ML5	Contaminant	1	9	1.40	N/A	0.0 (0.0)
KRT17	ML5	Contaminant	1	12	2.77	N/A	0.0 (0.0)
AHSG	ML5	Contaminant	1	14	3.88	0.0 (0.0)	N/A
KRT2	ML5	Contaminant	1	8	1.23	100.0 (0.0)	N/A
KRT78	ML5	Contaminant	1	7	1.34	100.0 (0.0)	N/A
AFM	ML5	Contaminant	1	12	1.98	100.0 (0.0)	100.0 (0.0)
AMEL	ML5	Enamel proteome	550	171	83.41	98.0 (0.7)	98.0 (0.7)
AMBN	ML5	Enamel proteome	288	191	42.73	99.7 (0.1)	98.7 (0.7)
ENAM	ML5	Enamel proteome	487	187	16.37	97.5 (0.6)	96.9 (1.0)
MMP20	ML5	Enamel proteome	10	49	10.14	100.0 (0.0)	N/A
AMTN	ML5	Enamel proteome	3	13	6.22	N/A	100.0 (0.0)
ALB	ML5	Enamel proteome	5	28	4.60	80.9 (0.0)	N/A
COL17A1	ML5	Enamel proteome	36	16	1.07	N/A	100.0 (0.0)
COL3A1	ML6	Contaminant	1	14	0.95	N/A	N/A
KRT17	ML6	Contaminant	1	12	2.77	N/A	0.0 (0.0)
AHSG	ML6	Contaminant	1	14	3.88	0.0 (0.0)	N/A
KRT2	ML6	Contaminant	1	8	1.23	100.0 (0.0)	N/A
AFM	ML6	Contaminant	1	12	1.98	100.0 (0.0)	100.0 (0.0)
AMEL	ML6	Enamel proteome	463	168	81.95	96.1 (1.5)	97.3 (0.9)
AMBN	ML6	Enamel proteome	331	193	43.18	99.0 (0.7)	98.4 (0.7)
ENAM	ML6	Enamel proteome	500	196	17.16	93.7 (1.6)	96.8 (0.9)
MMP20	ML6	Enamel proteome	8	45	9.32	100.0 (0.0)	100.0 (0.0)
AMTN	ML6	Enamel proteome	3	20	9.57	N/A	100.0 (0.0)
ALB	ML6	Enamel proteome	8	24	3.94	86.5 (0.0)	100.0 (0.0)
COL17A1	ML6	Enamel proteome	52	119	7.95	N/A	100.0 (0.0)

AHSG	S1113	Contaminant	1	14	3.88	0.0 (0.0)	N/A
KRT2	S1113	Contaminant	1	8	1.23	98.5 (0.0)	N/A
KRT78	S1113	Contaminant	1	7	1.34	100 (0.0)	N/A
AFM	S1113	Contaminant	1	12	1.98	100 (0.0)	100 (0.0)
AMEL	S1113	Enamel proteome	532	179	87.32	94.8 (2.2)	98.3 (0.6)
AMBN	S1113	Enamel proteome	333	216	48.32	99.9 (0.0)	98.6 (0.6)
ENAM	S1113	Enamel proteome	470	214	18.74	96.7 (1.5)	96.7 (1.0)
MMP20	S1113	Enamel proteome	4	30	6.21	100 (0.0)	N/A
AMTN	S1113	Enamel proteome	20	31	14.83	N/A	94.6 (5.3)
ALB	S1113	Enamel proteome	3	16	2.63	87.3 (4.1)	N/A
COL17A1	S1113	Enamel proteome	39	96	6.41	N/A	100 (0.0)
AFM	S1128	Contaminant	1	12	1.98	100.0 (0.0)	100.0 (0.0)
AMEL	S1128	Enamel proteome	380	177	86.34	100.0 (0.0)	99.3 (0.4)
AMBN	S1128	Enamel proteome	166	140	31.32	100.0 (0.0)	99.1 (0.8)
ENAM	S1128	Enamel proteome	270	186	16.29	91.7 (4.4)	95.0 (1.7)
MMP20	S1128	Enamel proteome	3	27	5.59	100.0 (0.0)	N/A
AMTN	S1128	Enamel proteome	2	12	5.74	N/A	100.0 (0.0)
ALB	S1128	Enamel proteome	3	15	2.46	100 (0.0)	N/A
COL17A1	S1128	Enamel proteome	7	34	2.27	N/A	100.0 (0.0)
COL3A1	S1177	Contaminant	2	21	1.43	100.0 (0.0)	N/A
AHSG	S1177	Contaminant	1	14	3.88	0.0 (0.0)	N/A
KRT2	S1177	Contaminant	1	8	1.23	100.0 (0.0)	N/A
KRT78	S1177	Contaminant	1	7	1.34	100.0 (0.0)	N/A
AFM	S1177	Contaminant	1	12	1.98	100.0 (0.0)	100.0 (0.0)
AMEL	S1177	Enamel proteome	434	177	86.34	99.2 (0.7)	98.0 (0.6)
AMBN	S1177	Enamel proteome	177	151	33.78	100.0 (0.0)	98.3 (1.0)
ENAM	S1177	Enamel proteome	316	173	15.15	97.0 (1.3)	94.6 (1.7)
MMP20	S1177	Enamel proteome	2	18	3.73	100.0 (0.0)	N/A
AMTN	S1177	Enamel proteome	2	19	9.09	N/A	100.0 (0.0)
ALB	S1177	Enamel proteome	5	24	3.94	100.0 (0.0)	100.0 (0.0)
COL17A1	S1177	Enamel proteome	5	26	1.74	N/A	N/A
COL3A1	S1181	Contaminant	5	37	2.52	N/A	N/A
KRT2	S1181	Contaminant	1	8	1.23	100.0 (0.0)	N/A

AFM	S1181	Contaminant	1	12	1.98	100.0 (0.0)	100.0 (0.0)
AMEL	S1181	Enamel proteome	340	163	79.51	100.0 (0.0)	98.3 (0.8)
AMBN	S1181	Enamel proteome	167	162	36.24	100.0 (0.0)	98.7 (0.9)
ENAM	S1181	Enamel proteome	242	171	14.97	96.9 (2.0)	93.0 (2.1)
MMP20	S1181	Enamel proteome	5	21	4.35	100.0 (0.0)	N/A
AMTN	S1181	Enamel proteome	36	36	17.22	N/A	98.0 (1.7)
ALB	S1181	Enamel proteome	39	81	13.30	100.0 (0.0)	100.0 (0.0)
COL17A1	S1181	Enamel proteome	13	60	4.01	N/A	100.0 (0.0)
COL1a1	S1181	Enamel proteome	110	264	18.03	89.8 (10.6)	100.0 (0.0)
COL1a2	S1181	Enamel proteome	18	64	4.69	100.0 (0.0)	N/A
KRT2	S2246	Contaminant	1	8	1.23	98.2 (0.0)	N/A
KRT78	S2246	Contaminant	1	7	1.34	100.0 (0.0)	N/A
AFM	S2246	Contaminant	1	12	1.98	100.0 (0.0)	100.0 (0.0)
AMEL	S2246	Enamel proteome	421	177	86.34	97.1 (1.1)	97.8 (0.8)
AMBN	S2246	Enamel proteome	210	157	35.12	99.9 (0.0)	98.1 (0.9)
ENAM	S2246	Enamel proteome	381	181	15.85	96.5 (1.4)	95.6 (1.3)
MMP20	S2246	Enamel proteome	7	41	8.49	100.0 (0.0)	N/A
AMTN	S2246	Enamel proteome	10	27	12.92	N/A	100.0 (0.0)
ALB	S2246	Enamel proteome	13	65	10.67	97.9 (2.2)	100.0 (0.0)
COL17A1	S2246	Enamel proteome	20	61	4.07	N/A	100.0 (0.0)
KRT17	S2249	Contaminant	1	12	2.77	N/A	0.0 (0.0)
AHSG	S2249	Contaminant	1	14	3.88	0.0 (0.0)	N/A
KRT2	S2249	Contaminant	1	8	1.23	0.0 (0.0)	N/A
KRT78	S2249	Contaminant	1	7	1.34	100.0 (0.0)	N/A
AFM	S2249	Contaminant	1	12	1.98	100.0 (0.0)	100.0 (0.0)
AMEL	S2249	Enamel proteome	408	168	81.95	97.4 (1.8)	98.0 (0.8)
AMBN	S2249	Enamel proteome	222	156	34.90	99.9 (0.1)	98.3 (1.0)
ENAM	S2249	Enamel proteome	345	174	15.24	96.3 (1.2)	94.8 (1.5)
MMP20	S2249	Enamel proteome	3	30	6.21	100.0 (0.0)	N/A
AMTN	S2249	Enamel proteome	9	18	8.61	N/A	N/A
ALB	S2249	Enamel proteome	9	50	8.21	100.0 (0.0)	100.0 (0.0)

COL17A1	S2249	Enamel proteome	35	76	5.08	N/A	100.0 (0.0)
COL3A1	S2254	Contaminant	13	68	4.64	N/A	N/A
KRT2	S2254	Contaminant	1	8	1.23	100.0 (0.0)	N/A
KRT78	S2254	Contaminant	1	7	1.34	100.0 (0.0)	N/A
AMEL	S2254	Enamel proteome	82	87	42.44	100.0 (0.0)	96.1 (2.4)
AMBN	S2254	Enamel proteome	46	85	19.02	100.0 (0.0)	100.0 (0.0)
ENAM	S2254	Enamel proteome	85	65	5.69	100.0 (0.0)	92.7 (3.5)
AMTN	S2254	Enamel proteome	5	10	4.78	N/A	100.0 (0.0)
ALB	S2254	Enamel proteome	20	32	5.25	100.0 (0.0)	100.0 (0.0)
COL17A1	S2254	Enamel proteome	38	93	6.21	N/A	100.0 (0.0)
COL1a1	S2254	Enamel proteome	599	603	41.19	96.6 (2.4)	96.8 (1.3)
COL1a2	S2254	Enamel proteome	159	415	30.38	100.0 (0.0)	100.0 (0.0)
KRT2	S2258	Contaminant	1	7	1.08	100.0 (0.0)	N/A
AFM	S2258	Contaminant	1	12	1.98	100.0 (0.0)	100.0 (0.0)
AMEL	S2258	Enamel proteome	382	177	86.34	98.2 (1.0)	98.1 (0.8)
AMBN	S2258	Enamel proteome	168	158	35.35	99.9 (0.1)	99.4 (0.4)
ENAM	S2258	Enamel proteome	277	184	16.11	94.7 (1.9)	93.4 (2.0)
MMP20	S2258	Enamel proteome	0	0	0.00	100.0 (0.0)	N/A
AMTN	S2258	Enamel proteome	9	23	11.00	N/A	100.0 (0.0)
ALB	S2258	Enamel proteome	2	28	4.60	100.0 (0.0)	100.0 (0.0)
COL17A1	S2258	Enamel proteome	19	51	3.41	N/A	100.0 (0.0)
AHSG	F8864	Contaminant	1	14	3.88	0.0 (0.0)	N/A
KRT78	F8864	Contaminant	1	7	1.34	100.0 (0.0)	N/A
AFM	F8864	Contaminant	1	12	1.98	100.0 (0.0)	100.0 (0.0)
AMEL	F8864	Enamel proteome	315	154	75.12	97.1 (2.4)	98.6 (0.7)
AMBN	F8864	Enamel proteome	91	144	32.21	99.9 (0.1)	98.6 (1.0)
ENAM	F8864	Enamel proteome	256	180	15.76	98.7 (0.8)	96.0 (1.6)
MMP20	F8864	Enamel proteome	5	41	8.49	100.0 (0.0)	N/A
AMTN	F8864	Enamel proteome	4	29	13.88	N/A	100.0 (0.0)
ALB	F8864	Enamel proteome	4	23	3.78	100.0 (0.0)	100.0 (0.0)
COL17A1	F8864	Enamel proteome	2	0	0.00	N/A	N/A
