# Supplementary figures and images for: Geometric morphometrics and paleoproteomics enlighten the paleodiversity of Pongo
Source: PLoS One. 2023 Dec 15;18(12):e0291308. doi: 10.1371/journal.pone.0291308 (PMC10723683; doi:10.1371/journal.pone.0291308)

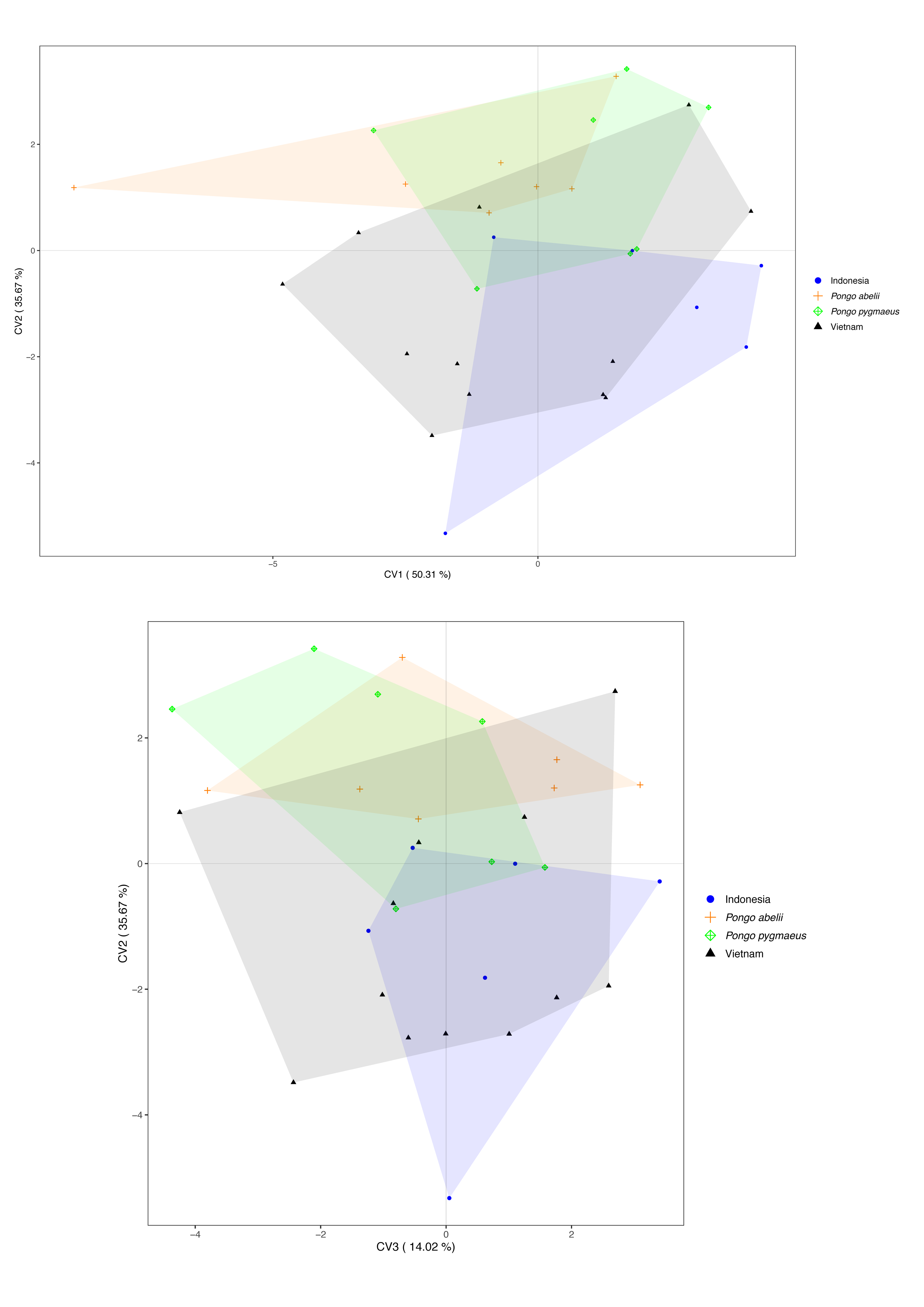

Supplement: S1 Fig — a Cross-validated CVA of the EDJbased on M1–M2. Jackknife cross-validation was performed using the same groups as in the CVA (Fig 1A) to validate group discrimination in Fig 1A. b Cross-validated CVA of the EDJ based on M3s. Jackknife cross-validation was performed using the same groups as in the CVA (Fig 1B) to validate group discrimination in Fig 1B. (ZIP) [file pone.0291308.s008.zip › S1b_Fig.tif]

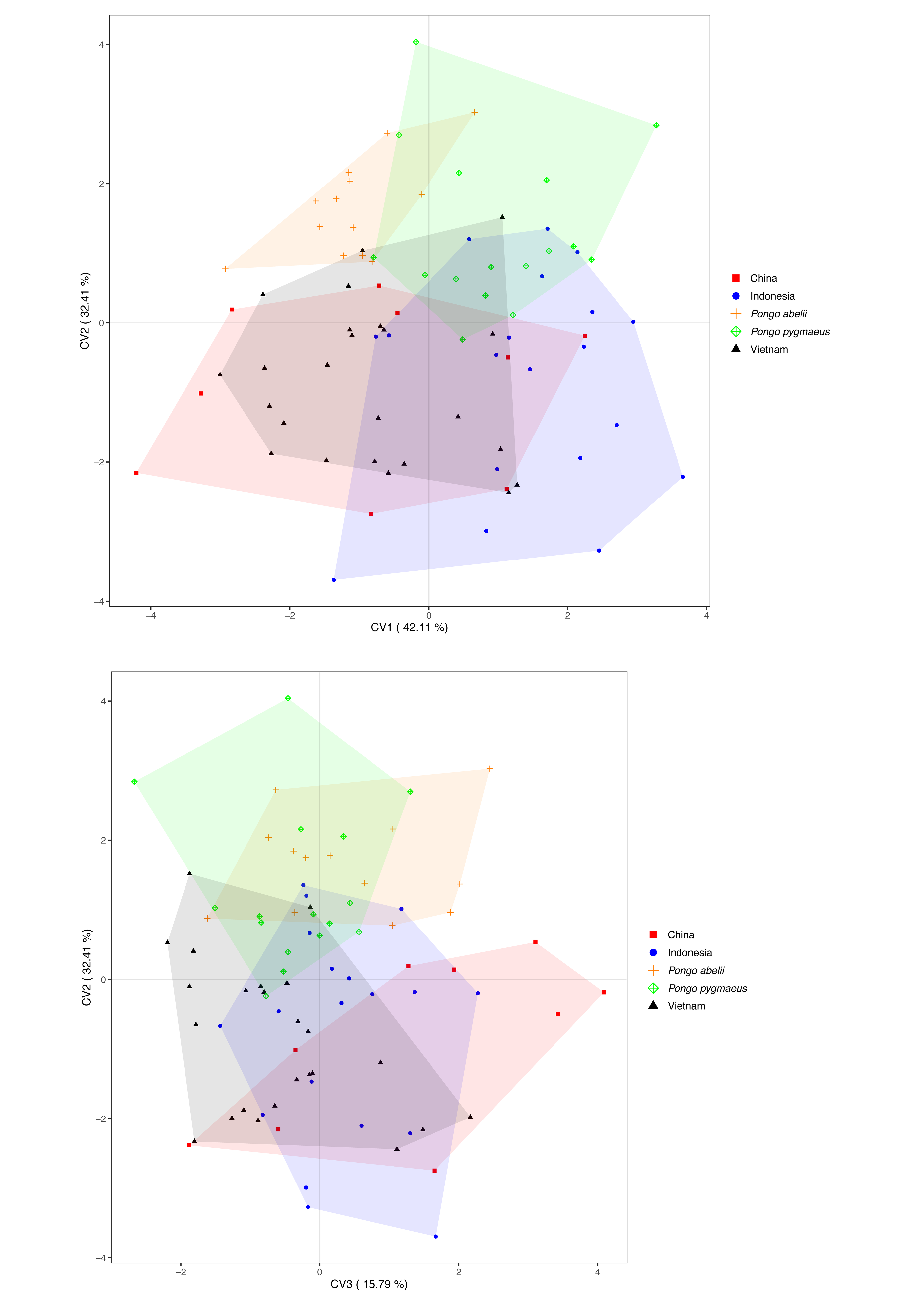

Supplement: S1 Fig — a Cross-validated CVA of the EDJbased on M1–M2. Jackknife cross-validation was performed using the same groups as in the CVA (Fig 1A) to validate group discrimination in Fig 1A. b Cross-validated CVA of the EDJ based on M3s. Jackknife cross-validation was performed using the same groups as in the CVA (Fig 1B) to validate group discrimination in Fig 1B. (ZIP) [file pone.0291308.s008.zip › S1a_Fig.tif]

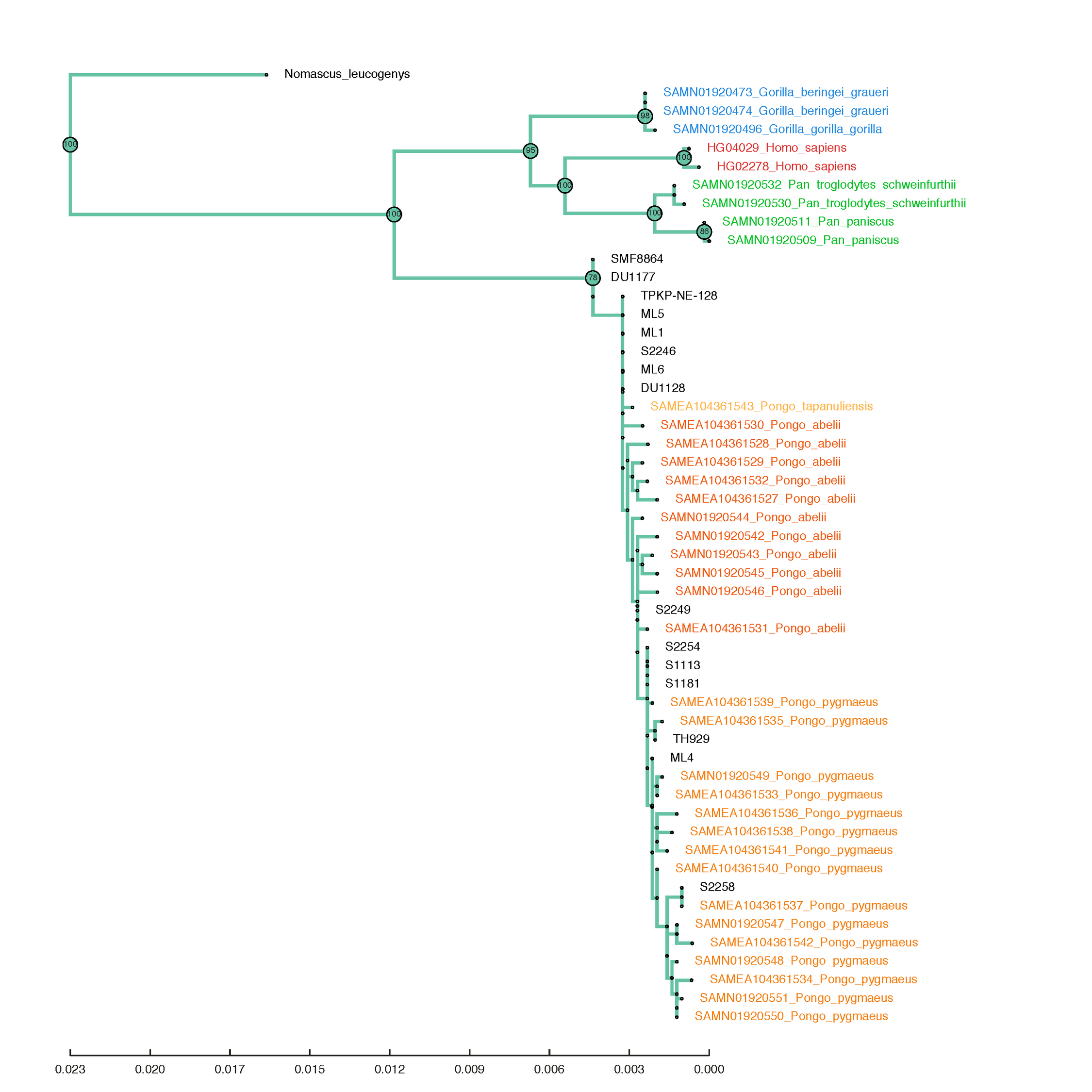

Supplement: S2 Fig — Maximum Likelihood phylogenetic tree created by using PhyML based on protein sequences of several great ape species (Gorilla gorilla, Gorilla beringei graueri, Pan paniscus, Pan troglodytes schweinfurthii, Pan troglodytes ellioti, Pongo pygmaeus, Pongo abelii, Pongo tapanuliensis and Homo sapiens) and fossil Pongo samples included. Nomascus leucogenys was added as an outgroup. In this tree both modern and fossil Pongo are placed within the same clade but with moderate support. Some substructure can be observed on the tree but without any concrete support. (TIF) [file pone.0291308.s009.tif]

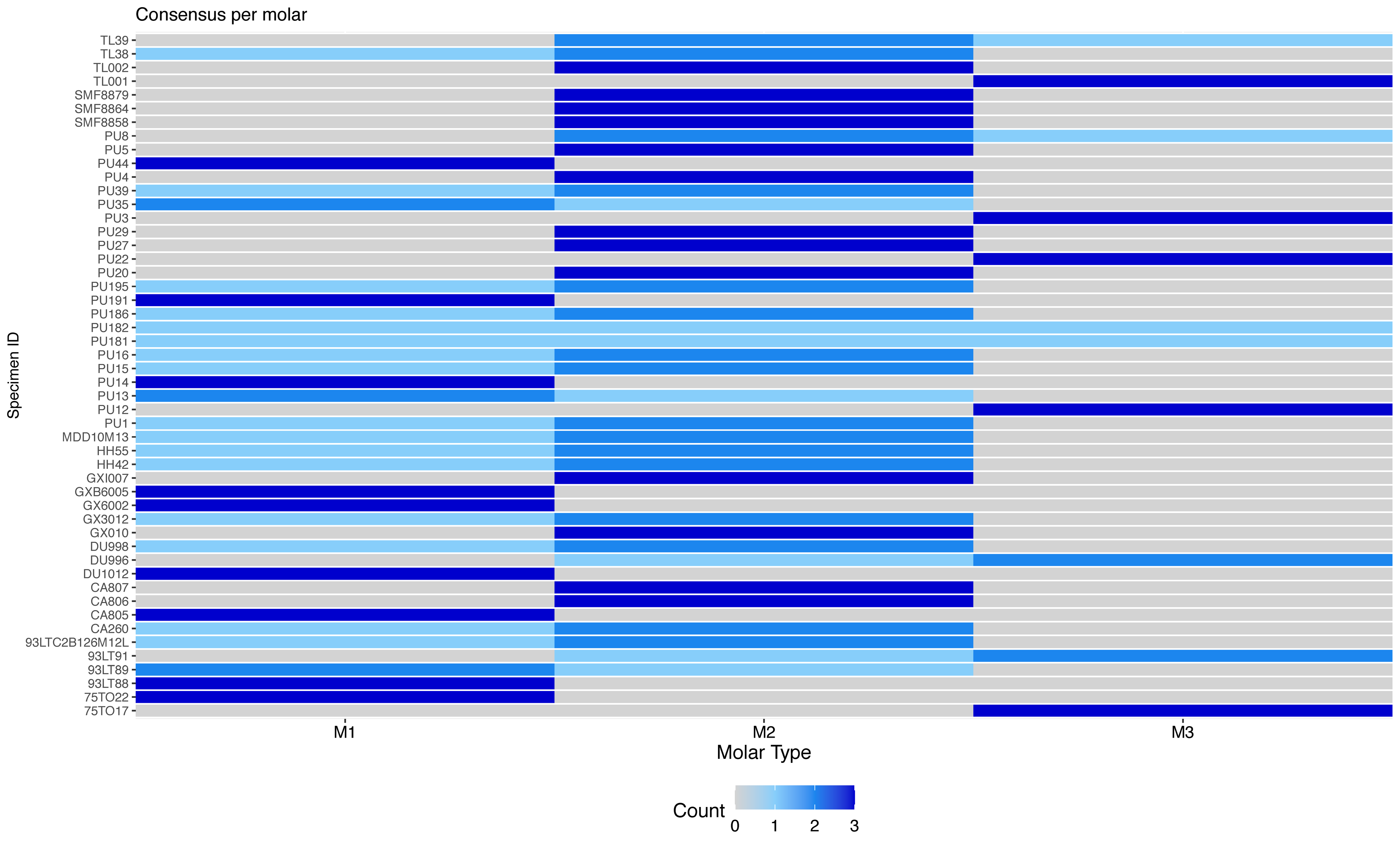

Supplement: S3 Fig — Heatmap of molar type assignments by three observers (JK, CZ, AMB) on a subsample of isolated Pongo fossil molars. Dark blue indicates consent by all 3 observers, medium blue indicates consent among 2 observers, light blue shows one observer assigned the molar to the specific position and light grey means no assignment. (TIF) [file pone.0291308.s010.tif]

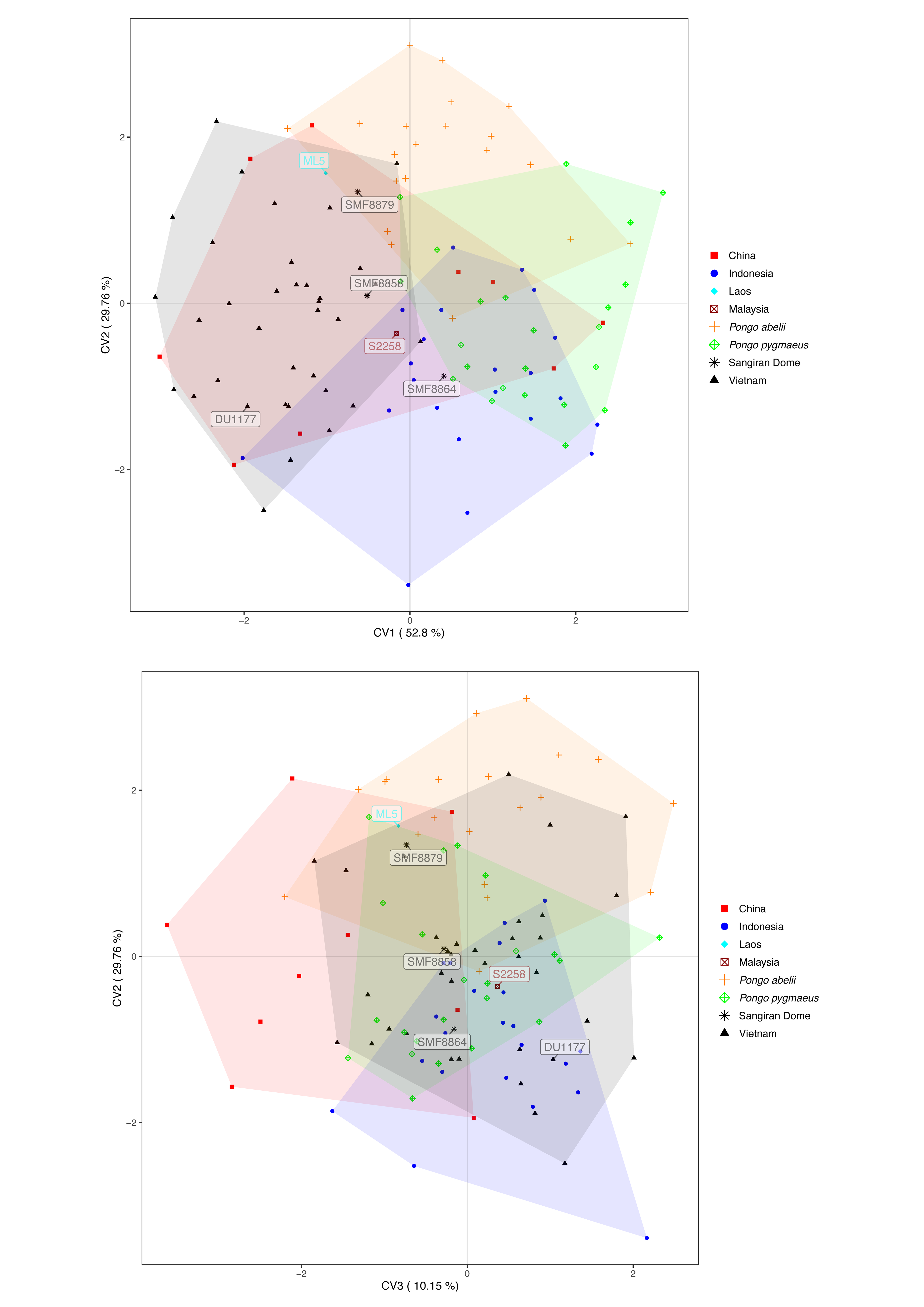

Supplement: S4 Fig — (TIF) [file pone.0291308.s011.tif]

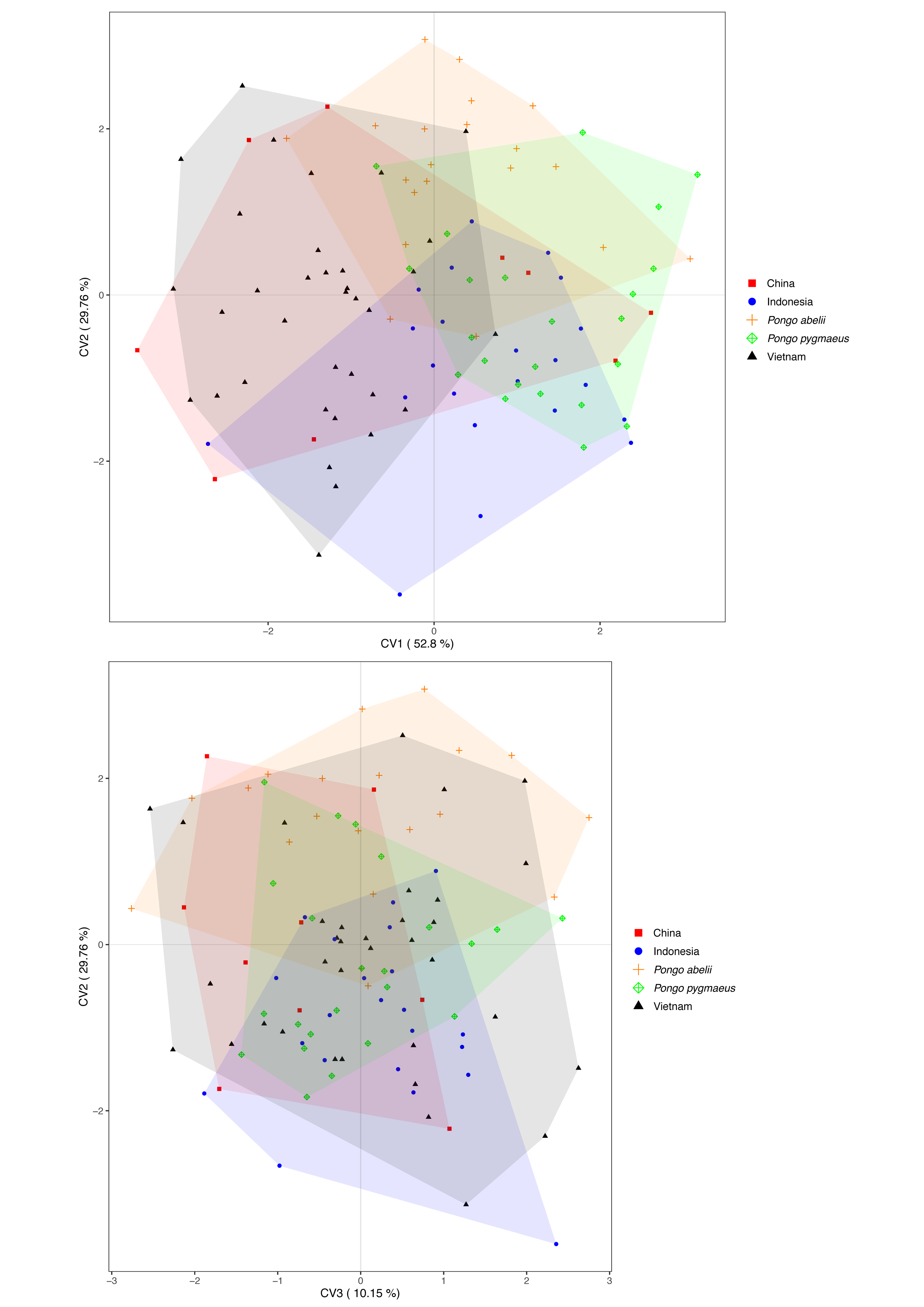

Supplement: S5 Fig — (TIF) [file pone.0291308.s012.tif]

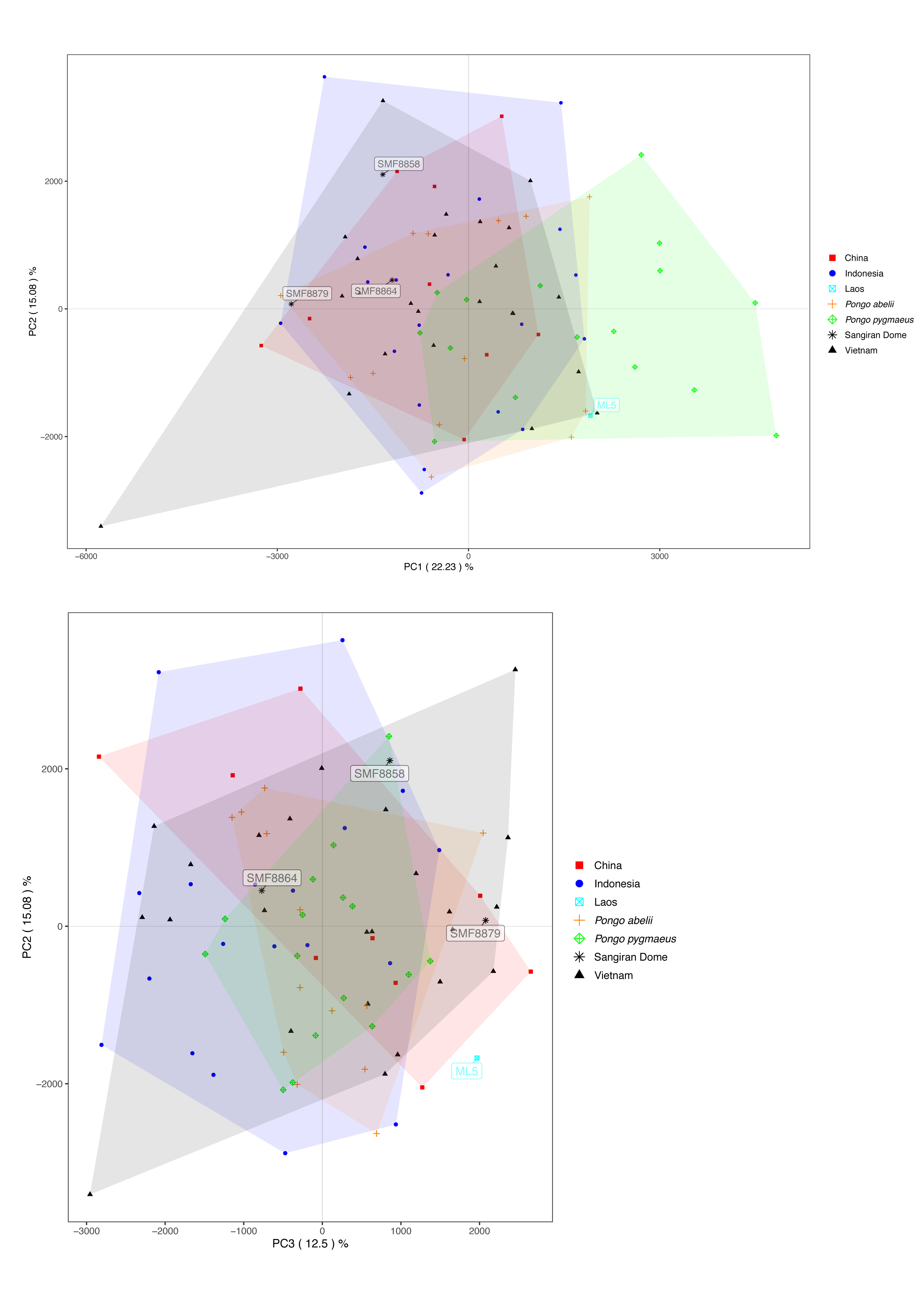

Supplement: S6 Fig — a PCA analyses of the EDJbased on M1–M2. b PCA analyses of the EDJ based on M3. c PCA analyses of the EDJ based on all molar positions. (ZIP) [file pone.0291308.s013.zip › S6a_Fig.tif]

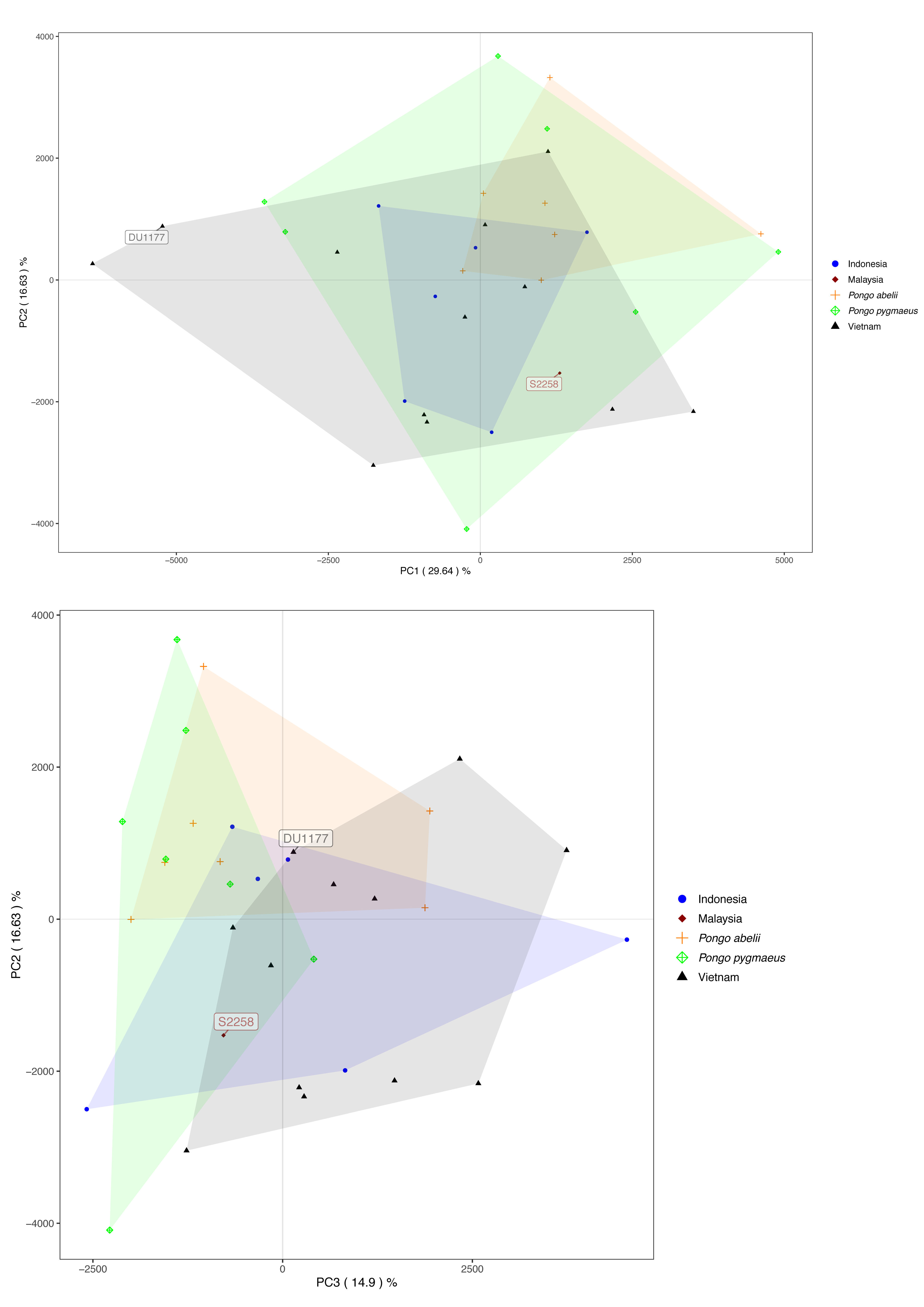

Supplement: S6 Fig — a PCA analyses of the EDJbased on M1–M2. b PCA analyses of the EDJ based on M3. c PCA analyses of the EDJ based on all molar positions. (ZIP) [file pone.0291308.s013.zip › S6b_Fig.tif]

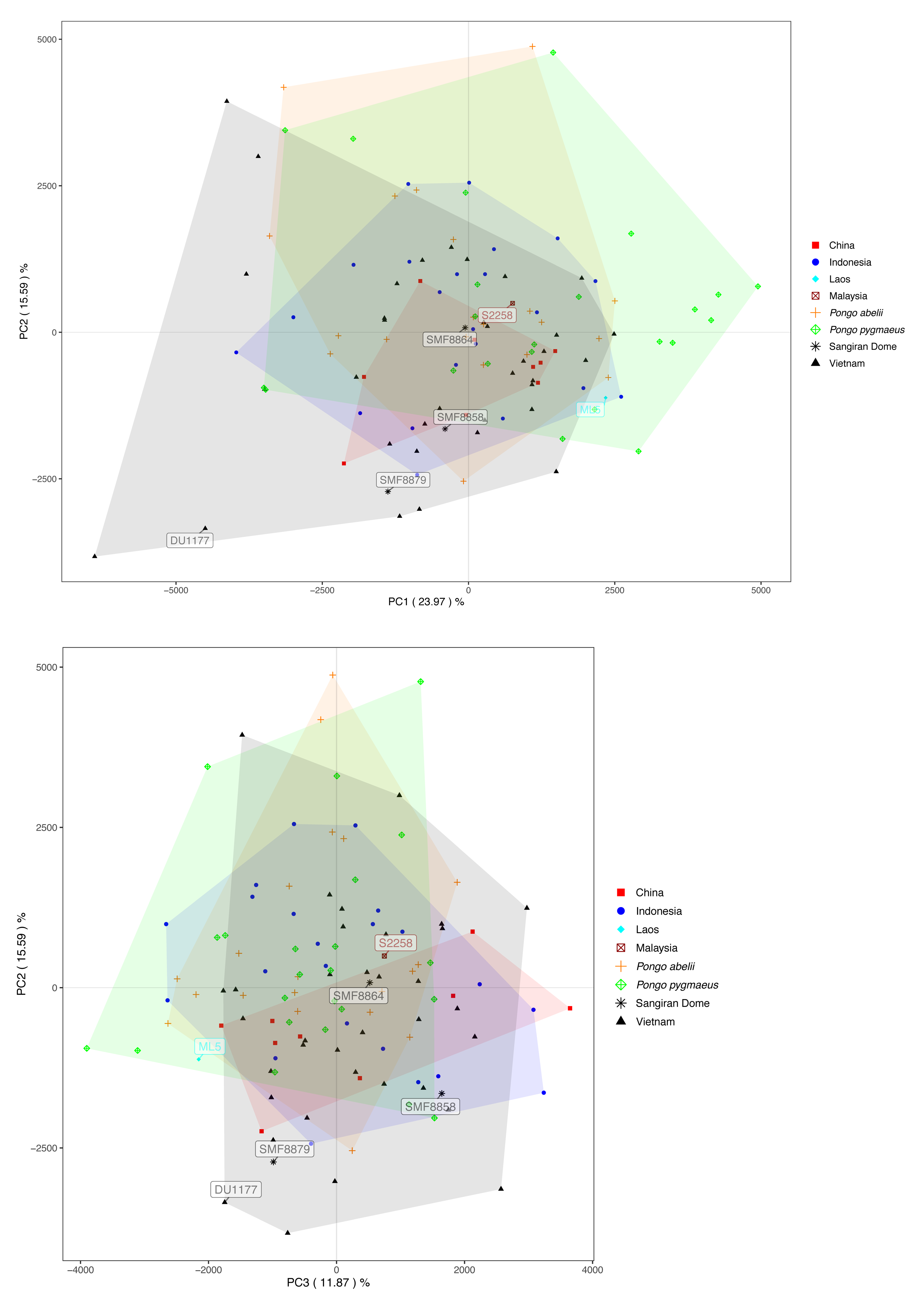

Supplement: S6 Fig — a PCA analyses of the EDJbased on M1–M2. b PCA analyses of the EDJ based on M3. c PCA analyses of the EDJ based on all molar positions. (ZIP) [file pone.0291308.s013.zip › S6c_Fig.tif]
