# Supplementary material for: Geometric morphometrics and paleoproteomics enlighten the paleodiversity of Pongo
Source: PLoS One. 2023 Dec 15;18(12):e0291308. doi: 10.1371/journal.pone.0291308 (PMC10723683; doi:10.1371/journal.pone.0291308)
Supplement: S1 File — (DOCX) [file pone.0291308.s014.docx]

**Supplementary Information**

**Palaeoproteomic Phylogenetic Analysis**

The assembled reference datasets are available in the ‘Raw_Reference_Dataset’ folder at <https://zenodo.org/record/8141962> and the reconstructed ancient proteins are available in the ‘Ancient Sequences’ folder.

The reference datasets were merged with the reconstructed proteins of the fossil specimens. A multiple sequence alignment (MSA) was generated by running Mafft on each protein fasta file. We used the default extension penalty of 0 but a modified lowered gap opening penalty of 0.5 due to the fact that ancient samples tend to have multiple gaps of missing data. For the same reason we also used the **--genafpair** option, as it is described in the mafft manual as “Suitable when large internal gaps are expected”. We chose to use the BLOSUM80 amino acid substitution matrix with the **--fmodel** option. The command used in Mafft is provided below:

mafft --ep 0 --op 0.5 --lop -0.5 --genafpair --maxiterate 20000 --thread {number threads} --bl 80 --fmodel samples.fa > samples_aligned.fa

The MSAs were corrected for the isobaric amino acids of Isoleucine (I) and Leucine (L). Positions in the alignment where either an I or an L was present in the recovered ancient proteins were marked. When all modern samples of the dataset were bearing either an I or an L, the amino acids of all ancient samples were switched to match that. When some of the modern samples were bearing an I and the rest were bearing an L, all Is were switched to Ls for that particular site and for all sequences in the MSA (modern or ancient). The final aligned and I/L corrected MSA for all proteins are available as fasta files in the ‘Alignments’ folder at <https://zenodo.org/record/8141962> .

The aligned and I/L corrected protein datasets were concatenated and then converted to the appropriate format for each phylogenetic software. If a sample in the alignment was missing a specific protein,the entire length of the missing protein was replaced by the missing symbol ‘?’ in the concatenation. These aligned and I/L corrected sequences were then used to generate the two phylogenetic trees using the two different software: MrBayes and PhyML. The commands for each phylogenetic analysis can be found below and all files of the analysis are also available in the ‘Phylogenetic_Analysis’ folder of <https://zenodo.org/record/8141962> .

####

- Commands for MrBayes

mb MrBayes_Commands.txt > Outputlog.txt;

Where ‘MrBayes_Commands.txt’ is a txt file that contains the command list provided below:

set autoclose=yes

execute CONCATINATED_o.nex

charset ENAM = 1-1151;

charset COL1A2 = 1152-2518;

charset AMELX = 2519-2723;

charset ALB = 2724-3332;

charset MMP20 = 3333-3815;

charset AMBN = 3816-4265;

charset AMTN = 4266-4484;

charset COL17A1 = 4485-6014;

partition BY_PROTEIN = 8: ENAM, COL1A2, AMELX, ALB, MMP20, AMBN, AMTN, COL17A1;

set partition=BY_PROTEIN;

prset aamodelpr = mixed;

mcmc nchains = 8 nruns=4 ngen = 1500000 samplefreq=100 printfreq=100 diagnfreq=1000;

sumt relburnin = yes burninfrac = 0.25;

sump;

quit;

The command in detail: The concatenated alignment was divided into partitions, one for each protein. Each partition used the same **‘mixed’** amino acid model of MrBayes. For the analysis 8 chains using 4 independent runs for 1.500.000 generations. The runs were all checked manually for convergence using Tracer (<https://beast.community/tracer>). All runs reported high ESS values and overlapping marginal densities between the runs (**CONCATINATED_o.nex.run*.p** files).

- Commands for PhyML:

phyml-mpi -i CONCATINATED_aln_e.phy -d aa -b 100 -m JTT -a e -s BEST -v e -o tlr -f m --rand_start --n_rand_starts 4 --r_seed $RAND --print_site_lnl --print_trace --no_memory_check

The command in detail: For the PhyML analysis we used the **JTT** model for 100 bootstraps along with a random start of 4 different trees and the **‘BEST’** search operation option. We also used the **-o tlr** option to optimize the tree topology (t), branch length (l) and rate parameters (r).

All output files of the MrBayes and the PhyML runs are available in the ‘Phylogenetic_Analysis\MrBayes’ and ‘Phylogenetic_Analysis\PhyML’ folders of <https://zenodo.org/record/8141962>.
